# Supplementary material for: Distribution of capsule and O types in Klebsiella pneumoniae causing neonatal sepsis in Africa and South Asia: A meta-analysis of genome-predicted serotype prevalence to inform potential vaccine coverage
Source: PLoS Med. 2026 Jan 12;23(1):e1004879. doi: 10.1371/journal.pmed.1004879 (PMC12810917; doi:10.1371/journal.pmed.1004879)
Supplement: S1 Table — Approval for the meta-analysis presented here was granted by the Observational/Interventions Research Ethics Committee of the London School of Hygiene and Tropical Medicine (ref #29931), and covers inclusion of data from the studies whose primary ethical approvals are listed in this table. (PDF) [file pmed.1004879.s001.pdf]

S1 Table. Ethics committees approving individual studies

Approval for the meta-analysis presented here was granted by the Observational / Interventions Research Ethics Committee of the London School of Hygiene and Tropical Medicine (ref #29931), and covers inclusion of data from the studies whose primary ethical approvals are listed in this table.

Study	Ethics Committees granting approval
**Baby GERMS-SA**	Human Research Ethics Committee of the University of the Witwatersrand (M190320). Approvals for the tier 2 surveillance study were received from each provincial research committee through registration on the National Health Research Database.
**BARNARDS**	Ethical Review Committee, Bangladesh Institute of Child Health, BICH-ERC-4/3/2015, 15/09/2015. Boston Children's Hospital, IRB-P00023058, 11/08/2016. Institutional Ethics Committee, National Institute of Cholera and Enteric Diseases and Institute of Post Graduate Medical Education and Research, A-I/2016-IEC, 17/11/2016. IPGMER Research Oversight Committee, Inst/IEC/2016/508, 04/11/2016. Kano State Hospitals Management Board, 8/10/1437AH, 13/07/2016. Health Research Ethics Committee (HREC), National Hospital, Abuja, NHA/EC/017/2015, 27/04/2015. Republic of Rwanda National Ethics Committee, No342/RNEC/2015, 10/11/2015. Stellenbosch University and Tygerberg Hospital, Research projects, Western Cape Government, N15/07/063, 04/12/2015 and 02/02/2016.
**KWTRP surveillance**	KEMRI Scientific and Ethics Review Unit, ref 281/4687, 17/4/2023. A research license was obtained from the National Commission for Science, Technology and Innovation, ref 527823, license no. NACOSTI/P/23/26005, 31/5/2023.
**GBS-COP**	University of the Witwatersrand, Human Research Ethics Committee (HREC), ref 181110, 24/4/2023.
**MLW Biobank**	University of Malawi College of Medicine Research Ethics Committee (COMREC) (P.11/18/2541).
**SPINZ**	Boston University Medical Center Institutional Review Board, USA (ref H-33473), 21/3/2016. Excellence in Research Ethics and Science (ERES) CONVERGE, Zambia (ref 2015-Jan-004), 1/3/2015.
**NIMBIplus**	NIMBI study: University of Pennsylvania IRB (ref 833786), 22/4/2020. Children's Hospital of Philadelphia Research Institute IRB (ref 19-016848), 25/7/2020. IRBs of Princess Marina Hospital IRB (PMH 2/11AII(372)), 20/3/2024 and the Health Research Development Committee (HRDC) in Botswana (ref HPRD 6/14/1), 15/2/2024.<br>SHARE study: University of Pennsylvania IRB (ref 851492), 9/6/2020. Princess Marina Hospital (ref PMH 2/2A(7)/201), 20/5/2022. University of Botswana IRB (ref UBR/RES/IRB/BIO/205), 11/5/2022. Health Research &

	Development Committee (HRDC) in Botswana (HPDME:13/18/1), 21/4/2022.
**MBIRA**	London School of Hygiene and Tropical Medicine HREC, ref 21236-4, latest amendment approved 27/3/2024. Korle Bu Teaching Hospital Institutional Review Board, 00097/2020, 18/11/2020; National Health Research Authority, 17/2/2020; Ministry of Science and Higher Education, 1.16/10.81/13, 24/2/2021; Western Cape Government, N20/02/072; National Institute for Medical Research, R.8a/Vol.IX/3575, 11/12/2020; Combined Research and Ethics Committee Swarm Hub research protocol v2, 2/11/2020.
**AKU**	Aga Khan University Ethics Review Committee, ref 2023-8485-25083, last renewed 13/5/2024.
**AIIMS**	Institute Ethics Committee, All India Institute of Medical Sciences, New Delhi, ref IEC-683/07.12.2018, RP-12/2018, 18/12/2018.
**CHRF**	Bangladesh Institute of Child Health HREC, ref BCH-ERC-02-02-2021, 3/2/2021.
