# Supplementary material for: Distribution of capsule and O types in Klebsiella pneumoniae causing neonatal sepsis in Africa and South Asia: A meta-analysis of genome-predicted serotype prevalence to inform potential vaccine coverage
Source: PLoS Med. 2026 Jan 12;23(1):e1004879. doi: 10.1371/journal.pmed.1004879 (PMC12810917; doi:10.1371/journal.pmed.1004879)
Supplement: S4 Table — Cumulative coverage estimates for the top 5, 10, 15, or 20 K loci (KL) ordered by cluster-adjusted global prevalence estimate. For each geographic location, the breakdown of isolate counts and corresponding coverage estimates is shown for subgroups defined by types of isolates (ESBL, extended-spectrum beta-lactamase; CP, carbapenemase-producing) or outcome of infection (fatal infections only). Coverage estimated from raw and cluster-adjusted counts (‘adj’) is shown as percentages, with 95% credible intervals in brackets. (PDF) [file pmed.1004879.s004.pdf]

S4 Table. Coverage estimates for KL set 1 (global top 20)

Cumulative coverage estimates for the top 5, 10, 15 or 20 K loci (KL) ordered by cluster-adjusted global prevalence estimate. For each geographic location, the breakdown of isolate counts and corresponding coverage estimates are shown for subgroups defined by types of isolates (ESBL, extended-spectrum beta-lactamase; CP, carbapenemase-producing) or outcome of infection (fatal infections only). Coverage estimated from raw and cluster-adjusted counts ('adj') are shown.

	**Counts**	**N**	**5 KL**	**10 KL**	**15 KL**	**20 KL**
**Global**	raw	1930	48.8 (46.0-51.7)	58.7 (55.6-61.9)	67.0 (63.6-70.4)	72.8 (69.3-76.4)
	adj	805	36.3 (32.4-40.3)	49.8 (45.3-54.6)	60.2 (55.2-65.4)	69.0 (63.6-74.6)
Fatal infections	raw	394	49.2 (43.1-55.7)	58.8 (51.8-66.1)	70.0 (62.3-77.9)	75.8 (67.8-84.2)
	adj	214	40.1 (31.5-49.7)	50.3 (40.5-61.2)	63.7 (52.5-76.3)	74.5 (62.3-88.1)
ESBL infections	raw	1747	50.8 (47.8-53.9)	60.8 (57.5-64.2)	69.5 (65.9-73.1)	75.0 (71.2-78.8)
	adj	683	38.2 (33.9-42.7)	52.3 (47.3-57.6)	63.5 (57.9-69.4)	72.1 (66.1-78.3)
CP infections	raw	525	40.9 (35.8-46.2)	51.3 (45.5-57.4)	65.7 (59.1-72.6)	71.7 (64.7-78.9)
	adj	228	24.0 (18.0-30.7)	41.5 (33.6-50.3)	56.6 (47.1-66.8)	68.5 (58.0-79.8)
**Eastern Africa**	raw	1119	55.9 (52.0-60.0)	65.8 (61.5-70.3)	70.7 (66.2-75.4)	75.7 (71.1-80.6)
	adj	367	42.1 (36.2-48.6)	55.5 (48.5-62.9)	63.8 (56.3-71.7)	71.0 (63.1-79.4)
Fatal infections	raw	237	56.4 (48.7-64.6)	64.5 (55.9-73.6)	70.2 (60.9-79.8)	74.2 (64.8-84.2)
	adj	100	45.5 (32.9-59.8)	54.4 (40.5-69.9)	64.9 (49.7-82.2)	73.6 (57.2-92.0)
ESBL infections	raw	1019	57.1 (52.9-61.3)	67.3 (62.7-72.0)	72.2 (67.4-77.0)	77.0 (72.1-82.0)
	adj	306	45.0 (38.2-52.2)	59.2 (51.2-67.7)	67.7 (59.2-76.7)	74.3 (65.4-83.7)
CP infections	raw	10	NA	NA	NA	NA
	adj	5	NA	NA	NA	NA
**Southern Africa**	raw	244	43.9 (36.5-51.9)	50.5 (42.4-59.0)	71.6 (62.3-81.6)	76.4 (66.6-86.8)
	adj	151	48.7 (39.1-59.4)	58.1 (47.6-69.5)	71.3 (59.8-84.3)	77.7 (65.4-91.4)
Fatal infections	raw	65	51.3 (31.9-74.1)	52.9 (33.1-75.9)	89.5 (64.4-100.0)	95.1 (69.1-100.0)
	adj	46	55.8 (31.4-85.8)	60.2 (34.8-90.7)	77.8 (48.0-100.0)	87.4 (55.1-100.0)
ESBL infections	raw	191	51.9 (42.6-61.8)	54.6 (45.0-64.7)	82.1 (70.3-94.1)	85.2 (73.1-97.6)
	adj	117	57.3 (44.7-71.0)	62.1 (48.9-76.7)	78.1 (62.9-94.1)	82.5 (66.9-99.3)
CP infections	raw	66	47.8 (30.9-67.4)	48.0 (31.1-67.6)	96.7 (74.2-100.0)	96.9 (74.4-100.0)
	adj	33	68.8 (38.4-100.0)	69.4 (38.8-100.0)	91.1 (55.7-100.0)	91.6 (56.1-100.0)
**Western Africa**	raw	76	28.4 (18.5-40.3)	38.2 (26.6-51.7)	44.5 (31.6-59.3)	50.5 (36.9-66.4)
	adj	55	29.8 (18.4-44.1)	37.8 (24.7-53.5)	46.6 (31.8-64.3)	54.9 (38.6-74.3)
Fatal infections	raw	36	19.6 (7.9-36.5)	40.4 (22.3-62.6)	51.0 (30.0-75.7)	60.5 (37.3-87.6)
	adj	28	27.8 (12.0-48.5)	38.5 (19.3-62.8)	52.1 (29.3-80.4)	64.4 (37.8-96.4)
ESBL infections	raw	62	32.8 (20.2-47.9)	44.8 (29.5-62.6)	52.2 (35.5-71.3)	59.0 (41.1-79.3)
	adj	43	34.9 (19.7-54.0)	43.5 (26.6-64.6)	54.1 (34.8-77.6)	63.4 (42.3-88.9)
CP infections	raw	30	5.4 (0.2-18.0)	5.7 (0.3-18.7)	6.3 (0.4-19.9)	6.7 (0.4-20.6)
	adj	17	9.4 (0.4-30.5)	10.3 (0.6-32.2)	11.5 (0.8-34.4)	12.2 (0.9-35.9)
**South Asia**	raw	491	38.3 (33.5-43.6)	49.9 (44.3-55.8)	59.6 (53.4-66.4)	67.8 (61.1-75.0)
	adj	232	20.8 (15.7-26.5)	38.5 (31.5-46.3)	50.5 (42.4-59.3)	63.6 (54.6-73.7)
Fatal infections	raw	56	35.8 (22.5-51.1)	50.7 (34.7-69.4)	66.8 (47.7-88.8)	78.1 (57.3-100.0)
	adj	40	25.7 (12.5-43.1)	42.6 (24.8-64.3)	59.7 (38.1-86.3)	75.2 (50.1-100.0)
ESBL infections	raw	475	39.0 (34.1-44.1)	51.0 (45.2-56.9)	61.1 (54.8-67.8)	68.8 (62.0-76.0)
	adj	217	20.7 (15.3-26.7)	39.8 (32.4-47.9)	52.7 (43.9-62.1)	65.8 (55.8-76.4)
CP infections	raw	419	41.9 (36.4-47.7)	54.0 (47.6-60.8)	65.4 (58.2-72.9)	72.3 (64.7-80.2)
	adj	173	20.3 (14.3-27.3)	40.8 (32.1-50.4)	56.1 (45.8-67.3)	70.1 (58.4-82.6)
