# Supplementary figures and images for: Distribution of capsule and O types in Klebsiella pneumoniae causing neonatal sepsis in Africa and South Asia: A meta-analysis of genome-predicted serotype prevalence to inform potential vaccine coverage
Source: PLoS Med. 2026 Jan 12;23(1):e1004879. doi: 10.1371/journal.pmed.1004879 (PMC12810917; doi:10.1371/journal.pmed.1004879)

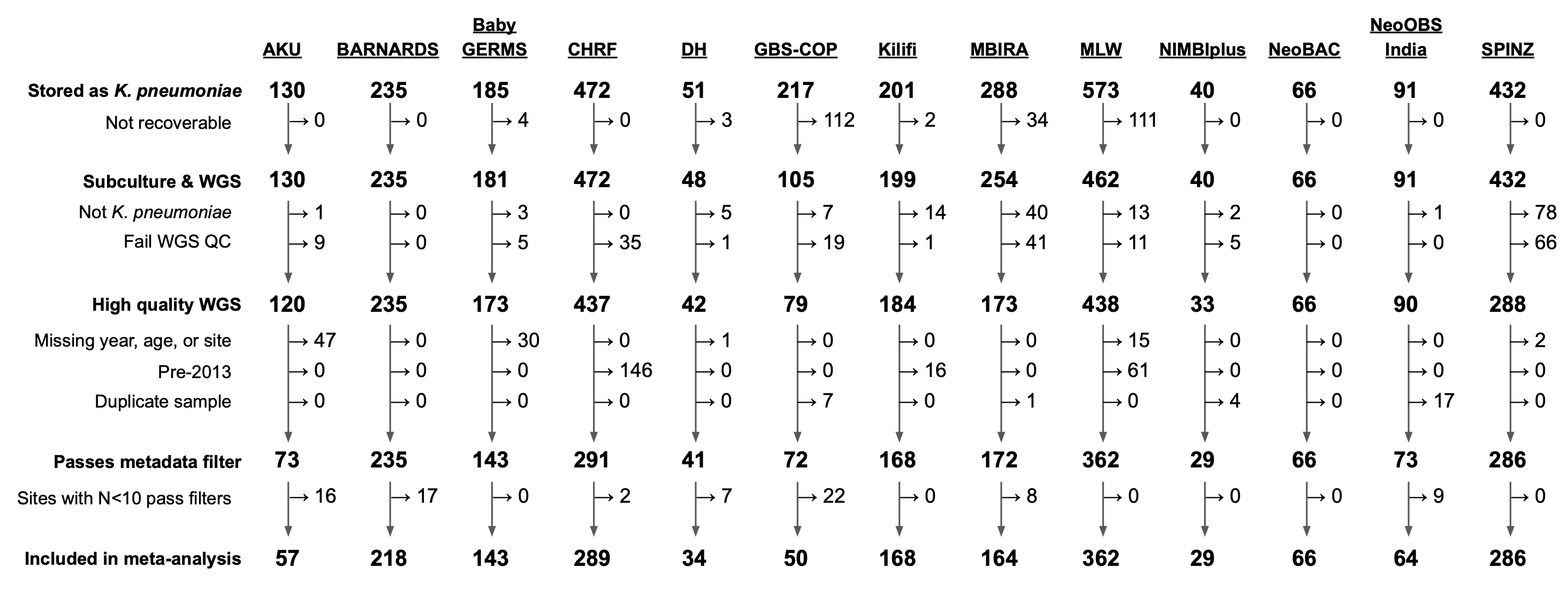

Supplement: S1 Fig — WGS, whole-genome sequencing; QC, quality control. (TIFF) [file pmed.1004879.s006.tiff]

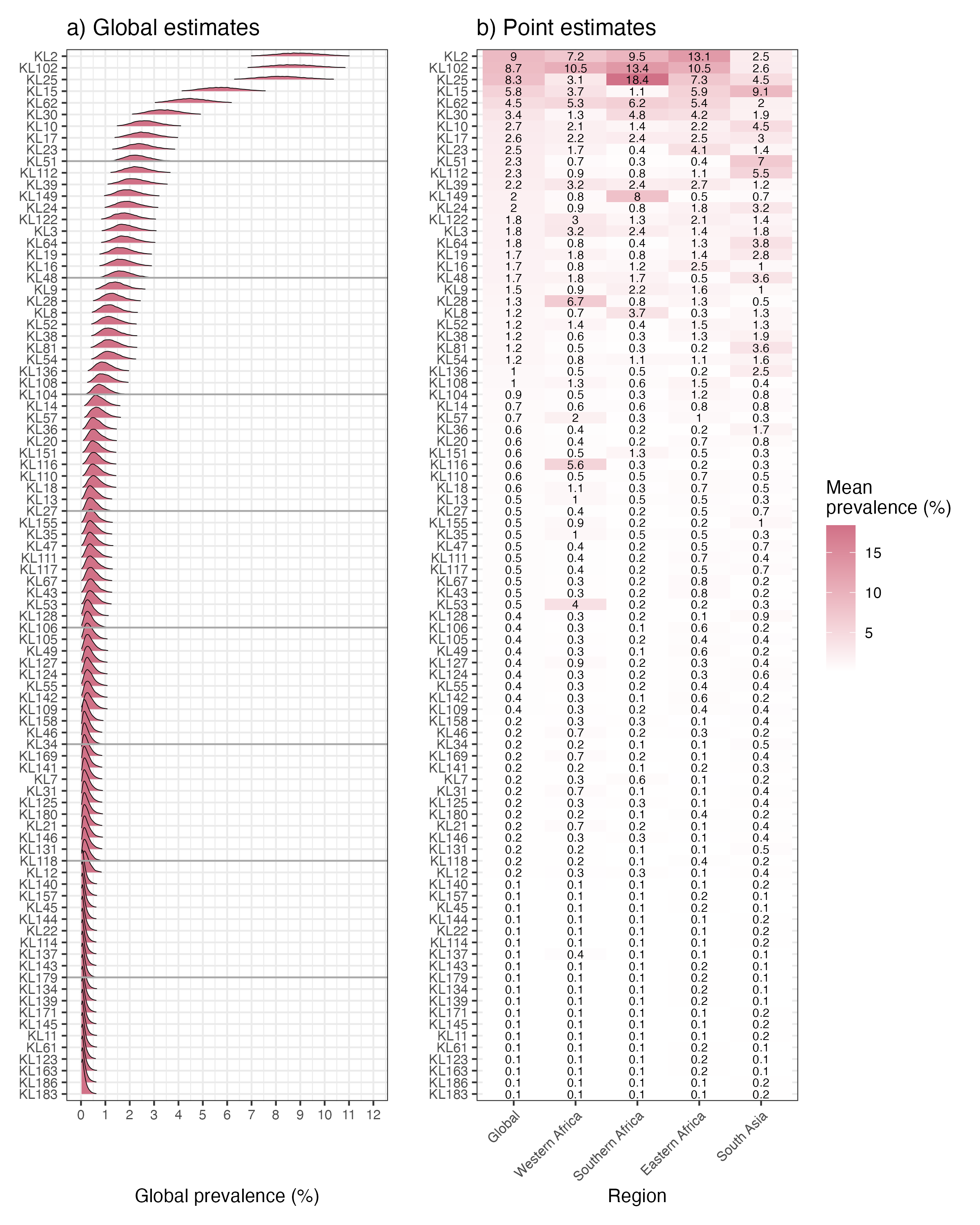

Supplement: S2 Fig — (a) Posterior density distribution for global (overall) prevalence estimates, modelled using cluster-adjusted counts per site, ordered by the mean point estimate. Horizontal lines indicate groups of 10 loci. (b) Regional prevalence estimates. Cells are coloured and labelled to indicate the cluster-adjusted regional mean prevalence estimates for each K locus in each region, according to the inset legend. (TIFF) [file pmed.1004879.s007.tiff]

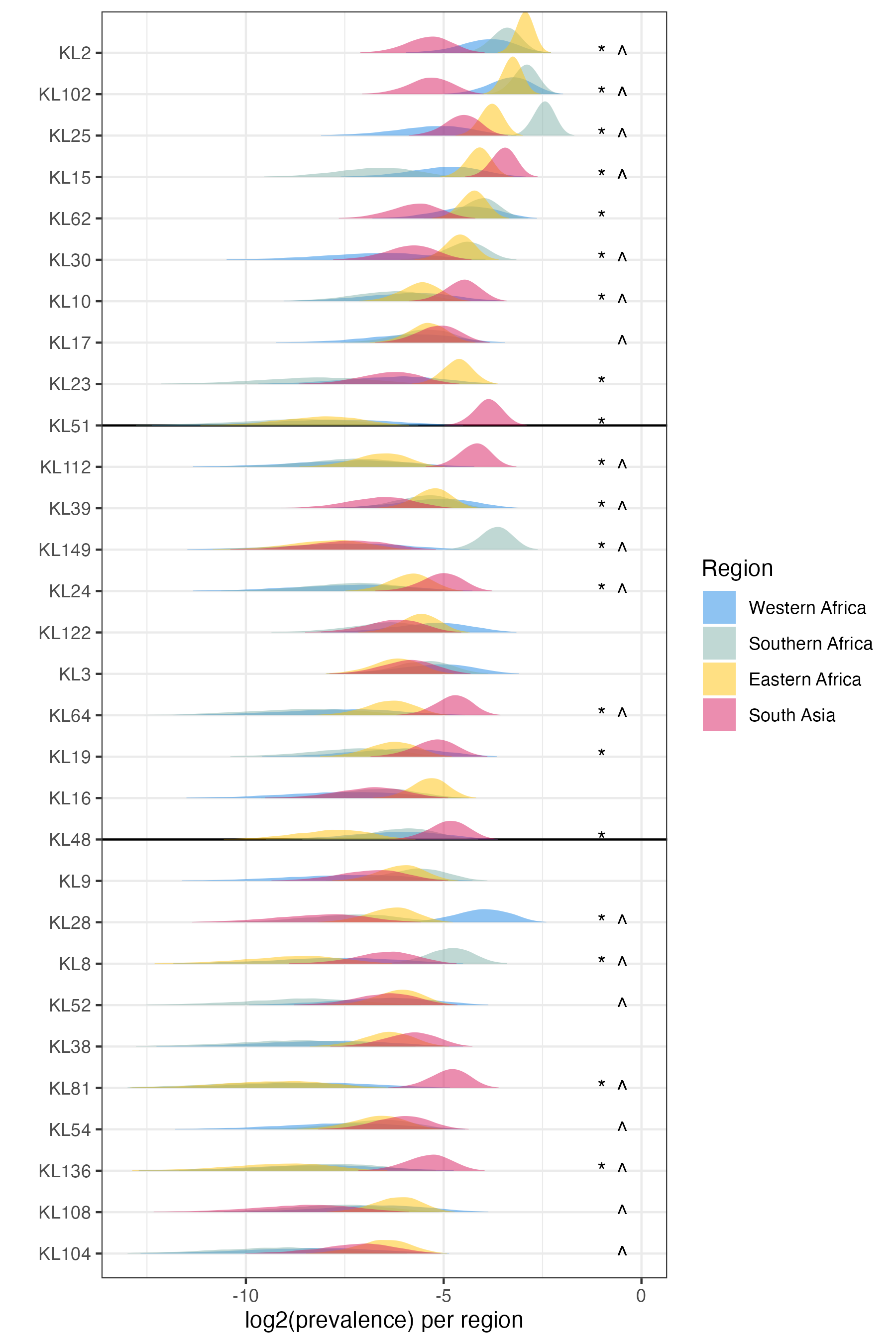

Supplement: S3 Fig — *Indicates K loci for which the median estimate per region differs by >2%, for at least one pair of regions. ^Indicates K loci for which region was a significant linear predictor in a logistic regression model. Horizontal lines indicate the top 10 and 20 global ranked loci. (TIFF) [file pmed.1004879.s008.tiff]

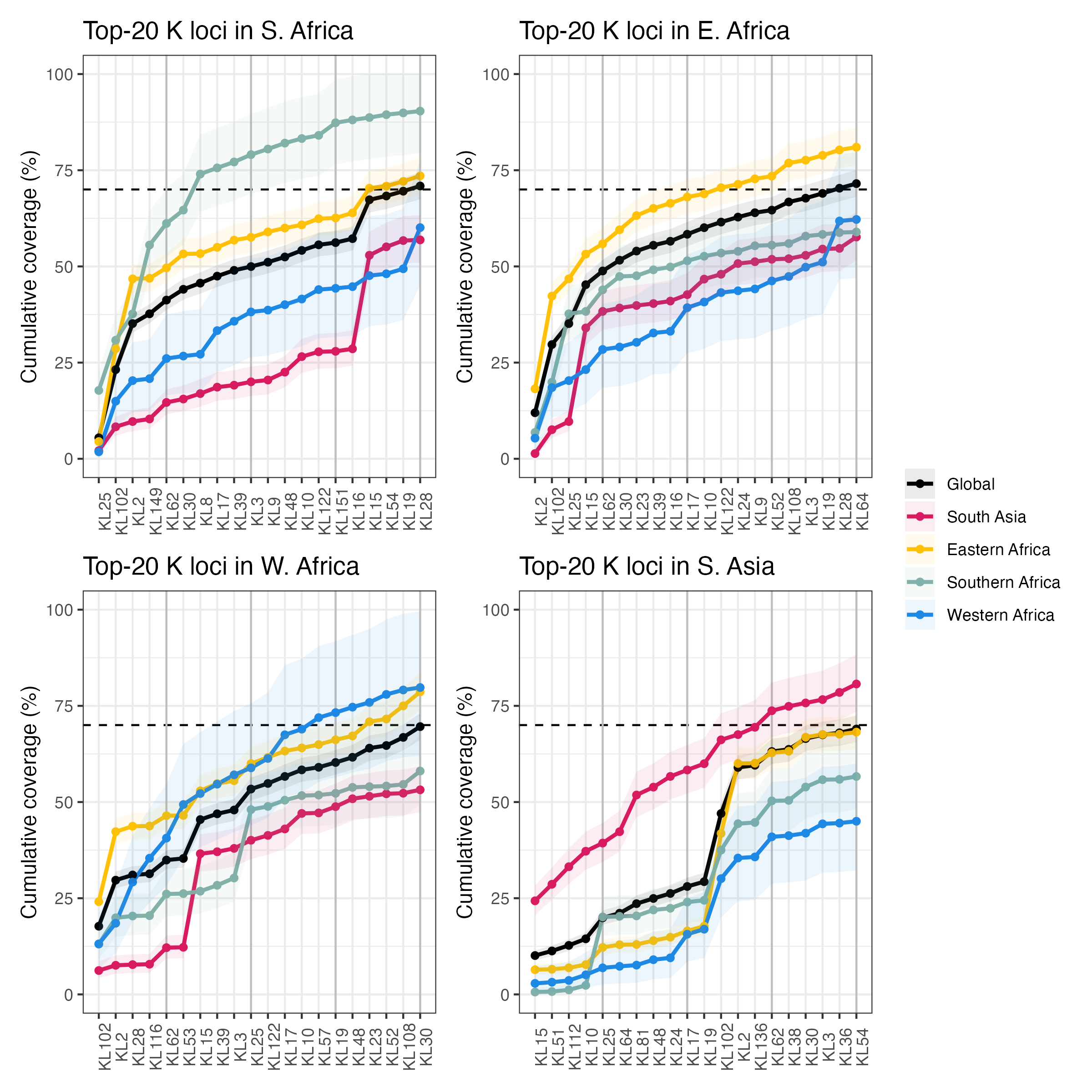

Supplement: S4 Fig — Each plot shows the modelled coverage in each region, using K loci ranked by cluster-adjusted counts in one region (indicated in the title of the plot). (TIFF) [file pmed.1004879.s009.tiff]

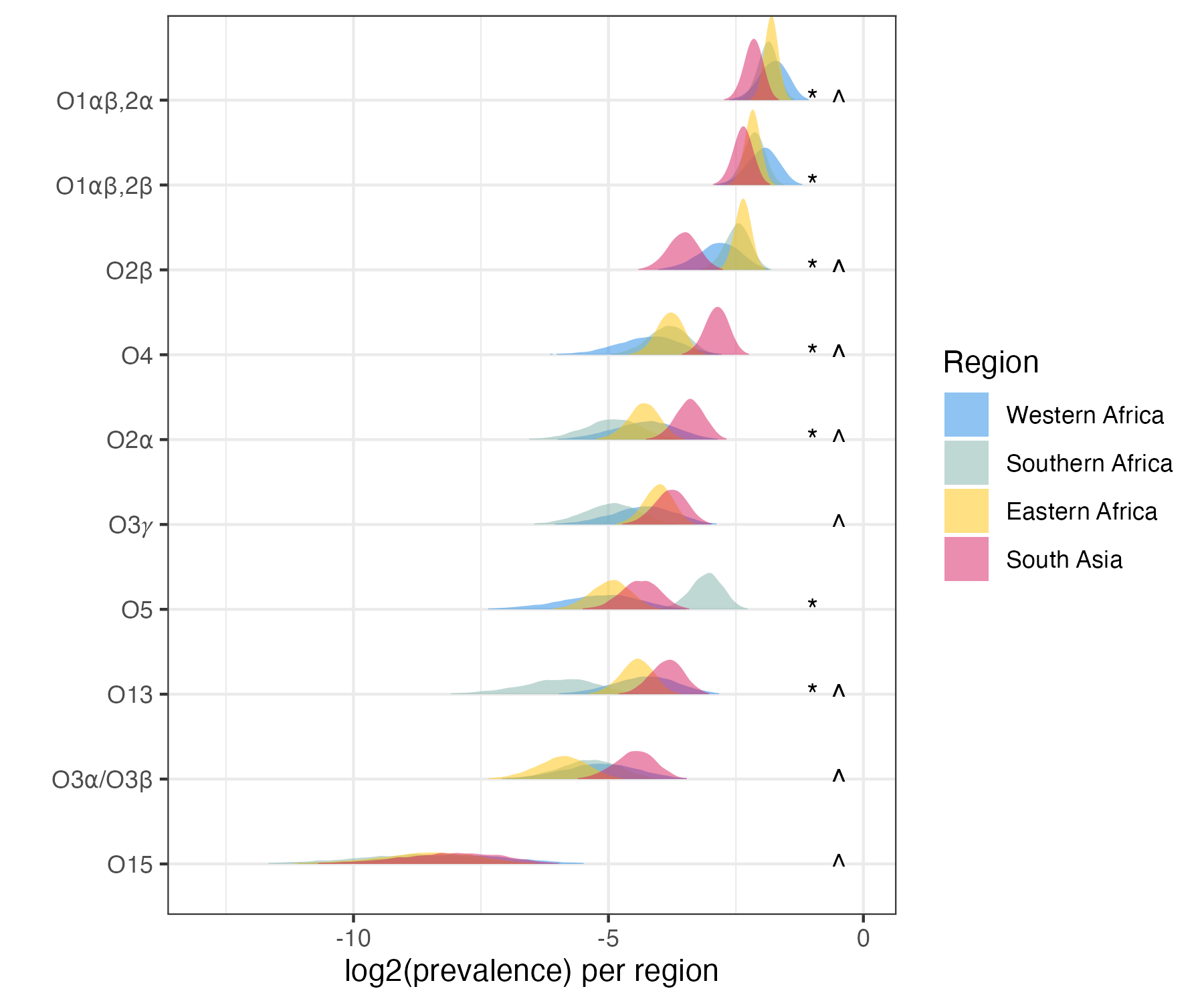

Supplement: S5 Fig — *Indicates O types for which the median estimate per region differs by >5%. ^Indicates O types for which region was a significant linear predictor in a logistic regression model. (TIFF) [file pmed.1004879.s010.tiff]

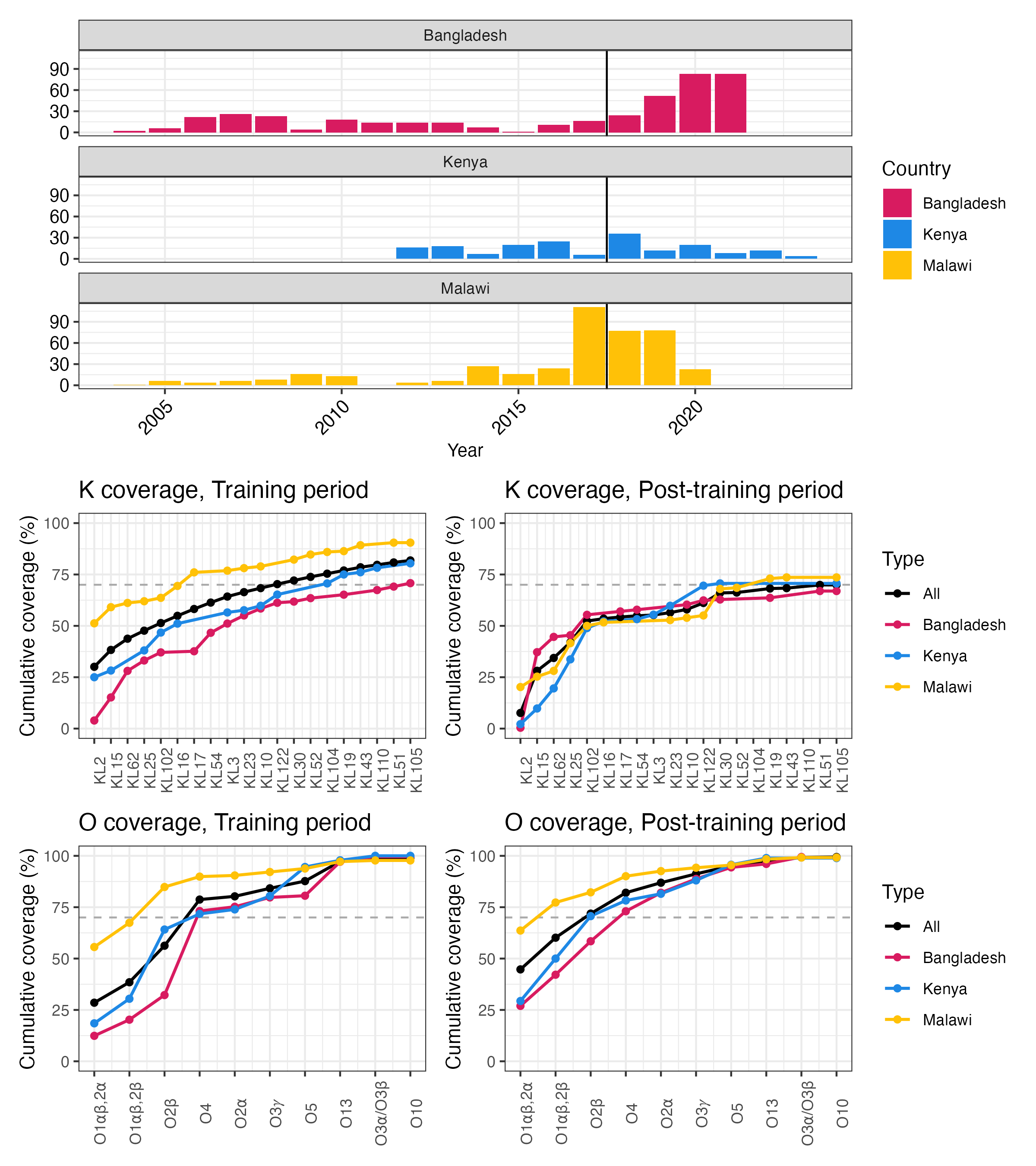

Supplement: S6 Fig — Three sites collected routine blood culture isolates for extended periods, providing an opportunity to assess whether a set of loci selected based on prevalence during one surveillance period (here, n = 512 samples obtained up to 2017 inclusive, labelled “Training”) could provide coverage of infections occurring in a later time period (here, n = 512 data after 2017, labelled “Post-training”). Note the coverage estimates shown reflect the crude proportion per surveillance site (or pooled across sites, labelled “All”), as opposed to Bayesian modelled estimates. (TIFF) [file pmed.1004879.s011.tiff]
