# Supplementary material for: Distribution of capsule and O types in Klebsiella pneumoniae causing neonatal sepsis in Africa and South Asia: A meta-analysis of genome-predicted serotype prevalence to inform potential vaccine coverage
Source: PLoS Med. 2026 Jan 12;23(1):e1004879. doi: 10.1371/journal.pmed.1004879 (PMC12810917; doi:10.1371/journal.pmed.1004879)
Supplement: S1 Appendix — (PDF) [file pmed.1004879.s012.pdf]

S1 Appendix: Genomic diversity.

S1.1 Appendix: Distribution of K loci and O types by country.

(a) Crude prevalence of K loci across the combined data set (n=1930 high quality genomes from 35 sites in 13 countries). All K loci identified in at least four isolates are included in the plot (n=53/87). Stacked barplot to the left shows the breakdown of common STs (accounting for >10 isolates each) amongst these K loci (coloured as per legend). **(b)** Distribution of K loci by country. Each point represents a single isolate, positioned in the grid to indicate the K locus (row) and country of origin (column), and coloured to indicate the O type (as per legend). Points are jittered around the x and y coordinates so that overlapping points (i.e. genomes of the same K locus and country) are visible.

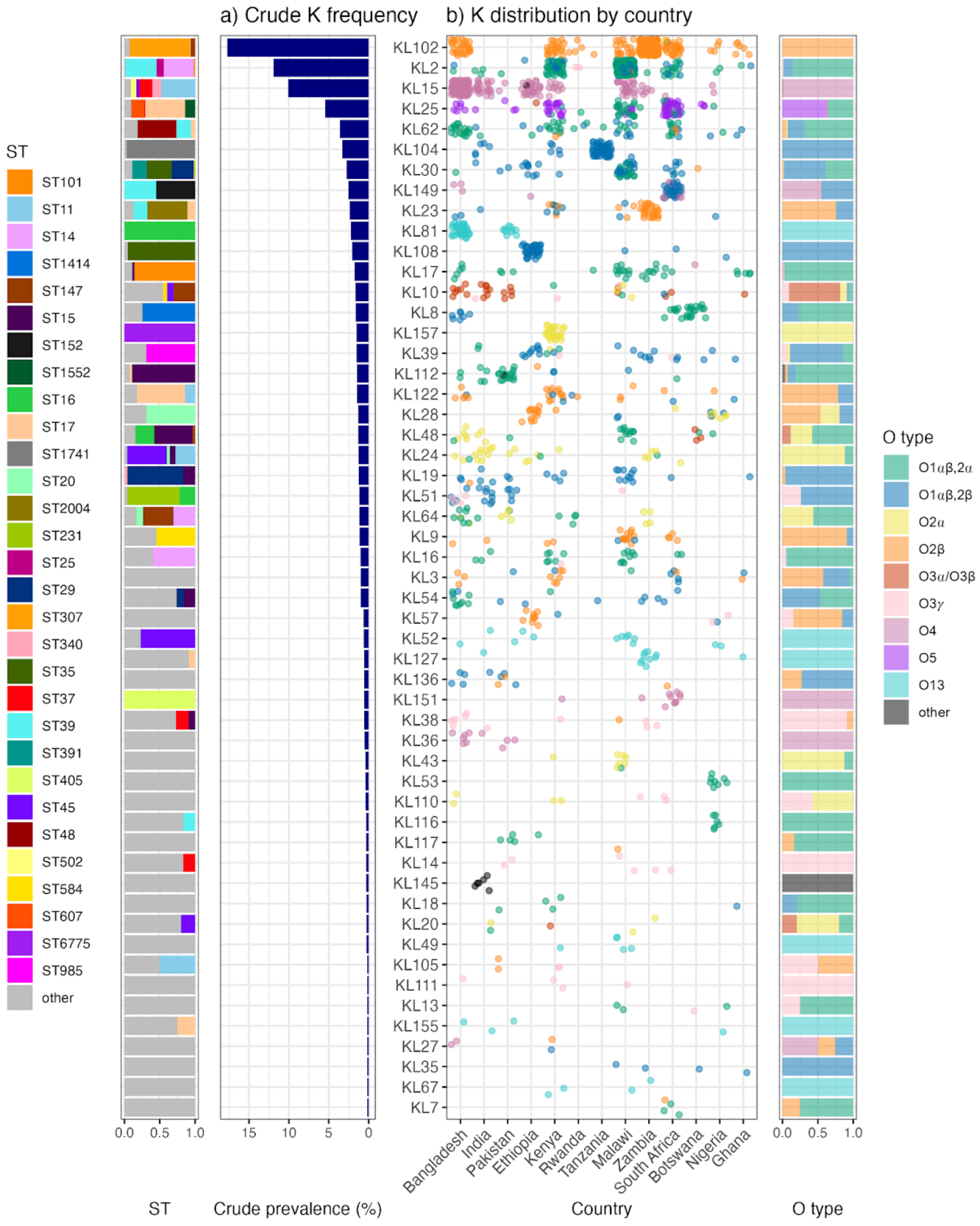

S1.2 Appendix: Distribution of clones per year per site.

Each panel shows the frequency, per year, of clones (defined as unique combinations of ST/K/O) detected at each study site (arrayed by region and country). Clones that were identified more than 15 times are coloured individually; the rest are grouped as ‘other’.

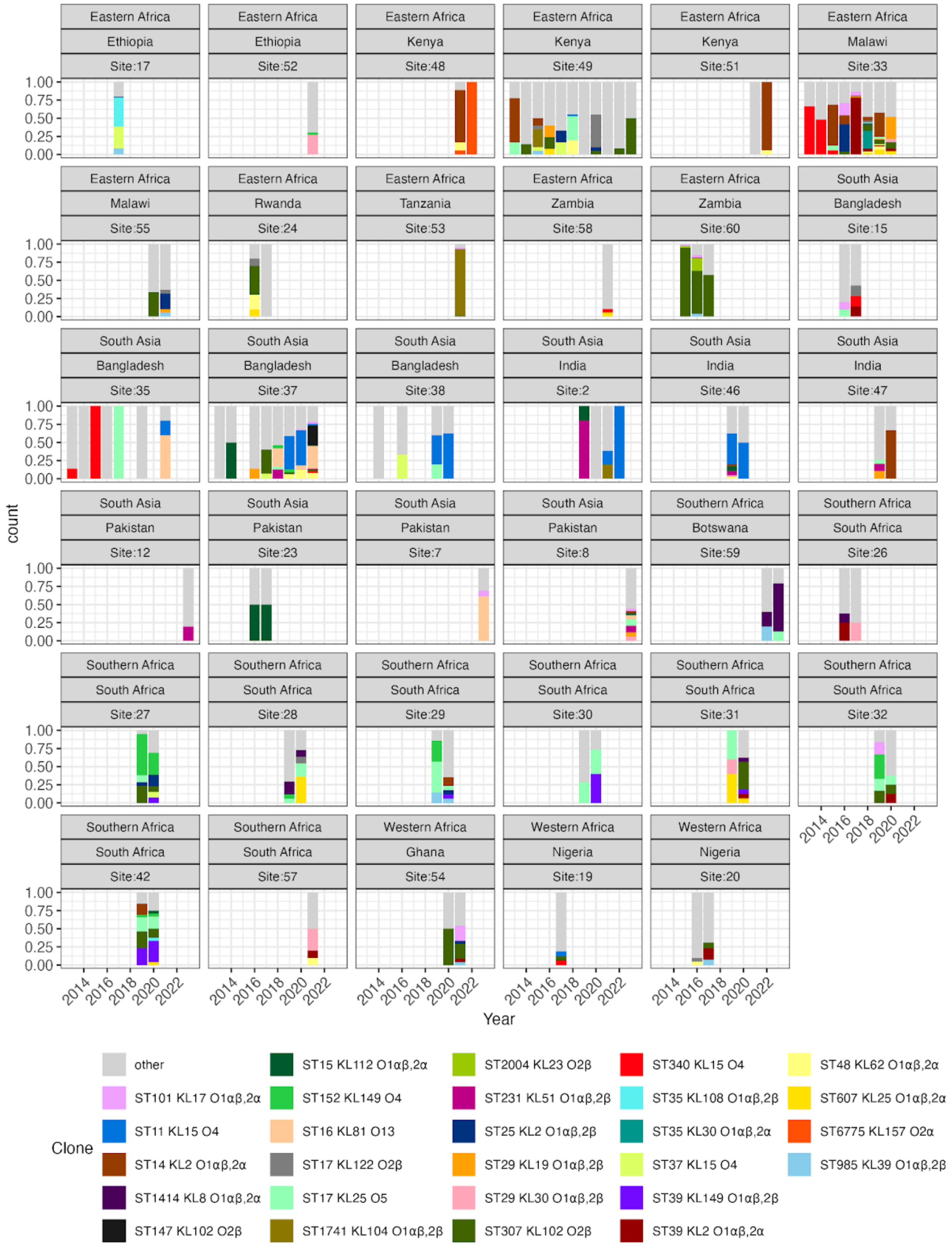

S1.3 Appendix: Crude cluster-adjusted annual frequency per K locus per region.

Bars show the crude frequency of each K locus per region, using cluster-adjusted counts. Regions are coloured by inset legend. These estimates are pooled across all available studies for that region and year, i.e. the total cluster-adjusted count of isolates per K locus per year in a given region, divided by the total number of unique clusters per year in that region. Plots are labelled with the K locus, and the rank of that K locus according to global Bayesian cluster-adjusted prevalence estimates. Regional variation highlighted in the text cannot be explained by differences in timing; e.g. KL51 were found consistently in Southern Asian isolates between 2018-2023, across 6 sites from 4 studies; but was not detected at all amongst 464 samples from Eastern Africa, 232 from Southern Africa and 26 from Western Africa in this period (KL81 showed similar). In Southern Africa, KL149 was detected at high prevalence in 2019 and 2020 (across 7 sites from three studies), but during this period was detected just twice amongst n=206 Southern Asian isolates and at all amongst n=136 Eastern African isolates.

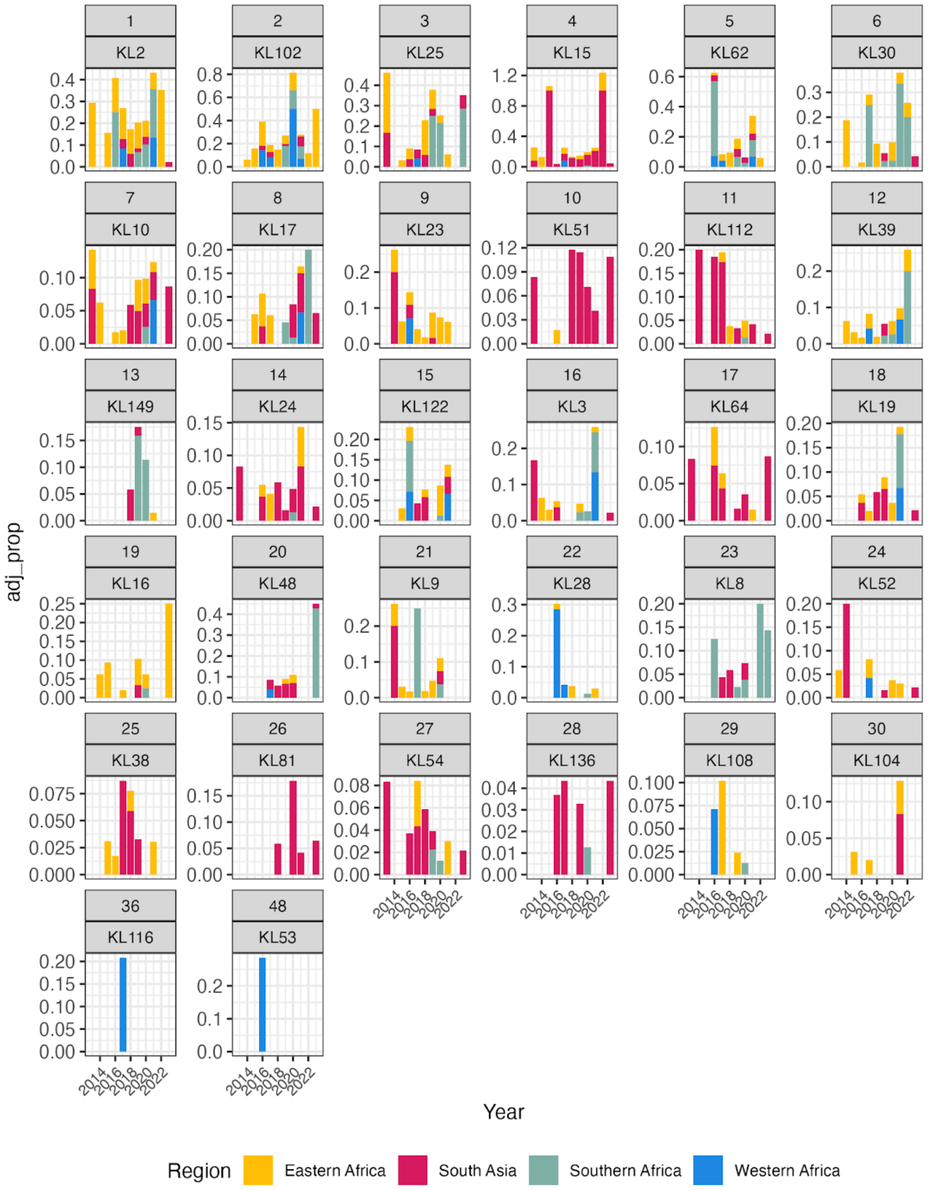

S1.4 Appendix: Crude cluster-adjusted annual frequency per O type per region.

Bars show the crude frequency of each O type per region, using cluster-adjusted counts. Regions are coloured by inset legend. These estimates are pooled across all available studies for that region and year, i.e. the total cluster-adjusted count of isolates per O type per year in a given region, divided by the total number of unique clusters per year in that region. Plots are labelled with the O type, and the rank of that O type according to global Bayesian cluster-adjusted prevalence estimates.

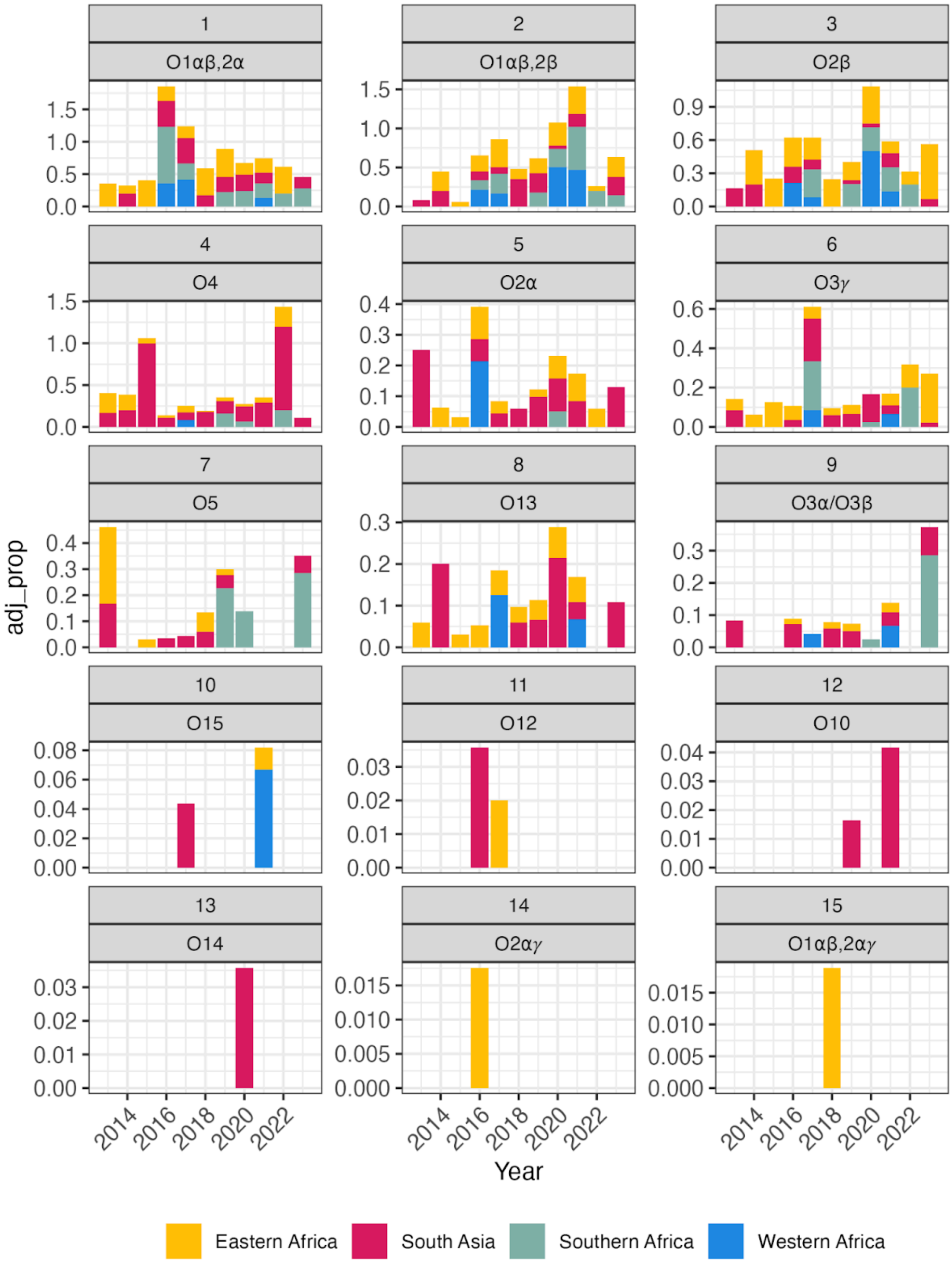

S1.5 Appendix: Region distribution of K and ST combinations associated with O types that demonstrate regional variation.

Each plot represents the full set of isolates carrying the O type indicated in the title; each point represents a single isolate with this O type in combination with a specific sequence type (ST, indicated by the row) and K locus (KL, indicated by the column).

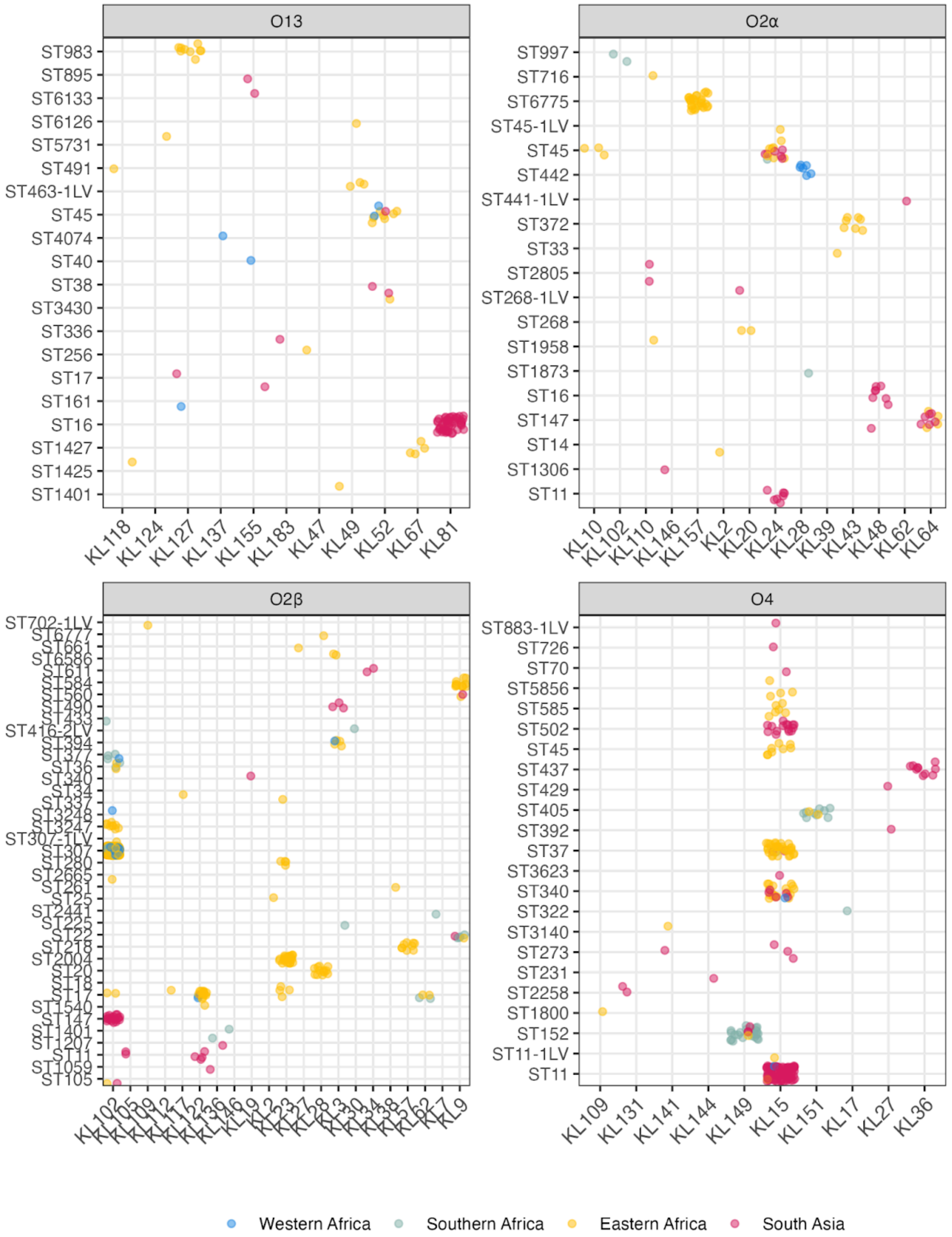

S1.6 Appendix: Definition of genetically inferred O types used in the analysis

The unit of analysis for predicted O antigen data in this study was predicted O type, defined from the Kaptive v fields ‘O_locus’ and ‘O_type’ as shown in this table. Due to small numbers we grouped O1α,2α and O1α,2β with their matching O1αβ subtypes, i.e. O1αβ,2α and O1αβ,2β as shown in the ‘Model group’ column.

Model group	O subtype	Kaptive v3.0 field: O_locus	Kaptive v3.0 field: O_type	n
O1αβ,2α	O1αβ,2α	O1/O2v1	O1ab	499
	O1α,2α	O1/O2v1	O1a	1
O1αβ,2β	O1αβ,2β	O1/O2v2	O1ab	342
	O1α,2β	O1/O2v2	O1a	1
O1αβ,2αγ	O1αβ,2αγ	O1/O2v3	O1ab	4
O2α	O2α	O1/O2v1	O2a	99
O2β	O2β	O1/O2v2	O2afg	471
O2αγ	O2αγ	O1/O2v3	O2a	1
O3α/O3β	O3α/O3β	O3/O3a	O3/O3a	35
O3γ	O3γ	O3b	O3b	54
O4	O4	O4	O4	251
O5	O5	O5	O5	68
O10	O10	OL103	OL103	7
O12	O12	O12	O12	2
O13	O13	OL13	O13	91
O14	O14	OL102	OL102	1
O15	O15	OL104	OL104	3
