# Supplementary material for: Distribution of capsule and O types in Klebsiella pneumoniae causing neonatal sepsis in Africa and South Asia: A meta-analysis of genome-predicted serotype prevalence to inform potential vaccine coverage
Source: PLoS Med. 2026 Jan 12;23(1):e1004879. doi: 10.1371/journal.pmed.1004879 (PMC12810917; doi:10.1371/journal.pmed.1004879)

S2 Appendix: Sensitivity analyses.

S2.1 Appendix: Sensitivity of K locus global ranks to study inclusion.

Plots show the results of a leave-one-out analysis. In the first matrix, each column shows the global (overall) ranking of K loci, based on mean prevalence estimates modelled from cluster-adjusted counts, including either all studies (column 1) or all-but-one study (other columns). Each column is labelled by the study that was excluded from the meta-analysis. Cells are coloured (as per inset legend) to illustrate the rank of each K locus (row) in each meta-analysis (column). All K loci ranked in the top-20 for any meta-analysis are included. In the second matrix, cells show the coverage of the global top-20 from the full analysis.

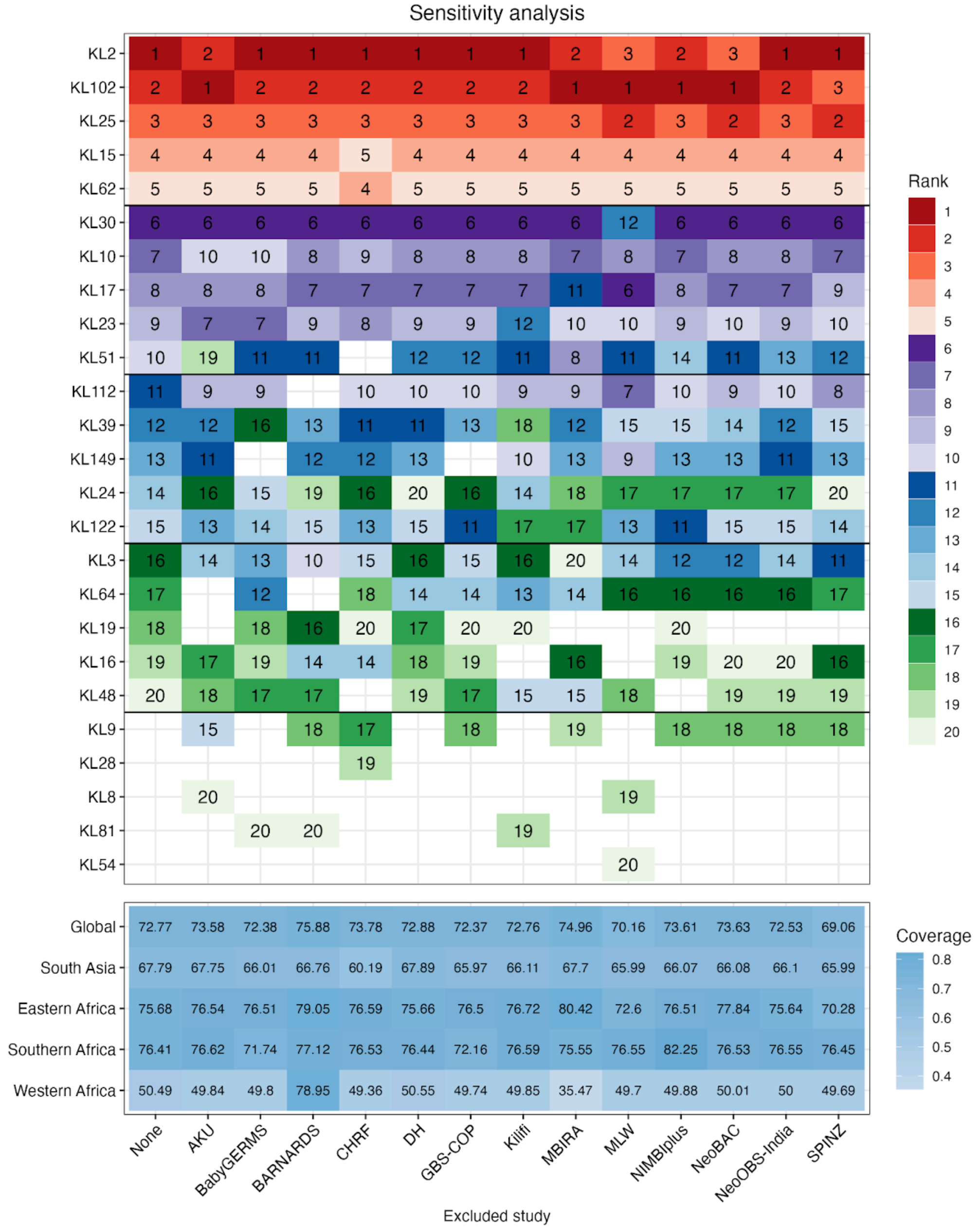

S2.2 Appendix: Sensitivity of O type global ranks to study inclusion.

Plots show the results of a leave-one-out analysis. Top matrix shows the results of a leave-one-out analysis. Each column shows the global (overall) ranking of O types, based on mean prevalence estimates modelled from cluster-adjusted counts, including either all studies (column 1) or all-but-one study (other columns). Each column is labelled by the study that was excluded from the meta-analysis. Cells are coloured (as per inset legend) to illustrate the rank of each O type (row) in each meta-analysis (column). Only the top nine O types are shown (the other six loci are excluded as they were rarely observed: O1αβ,2αγ, n=4; O2αγ, n=1; O12, n=2; O10, n=7; O14, n=1; O15, n=2). In the matrices below, cells show the coverage of the global top-4 or top-5 from the full analysis.

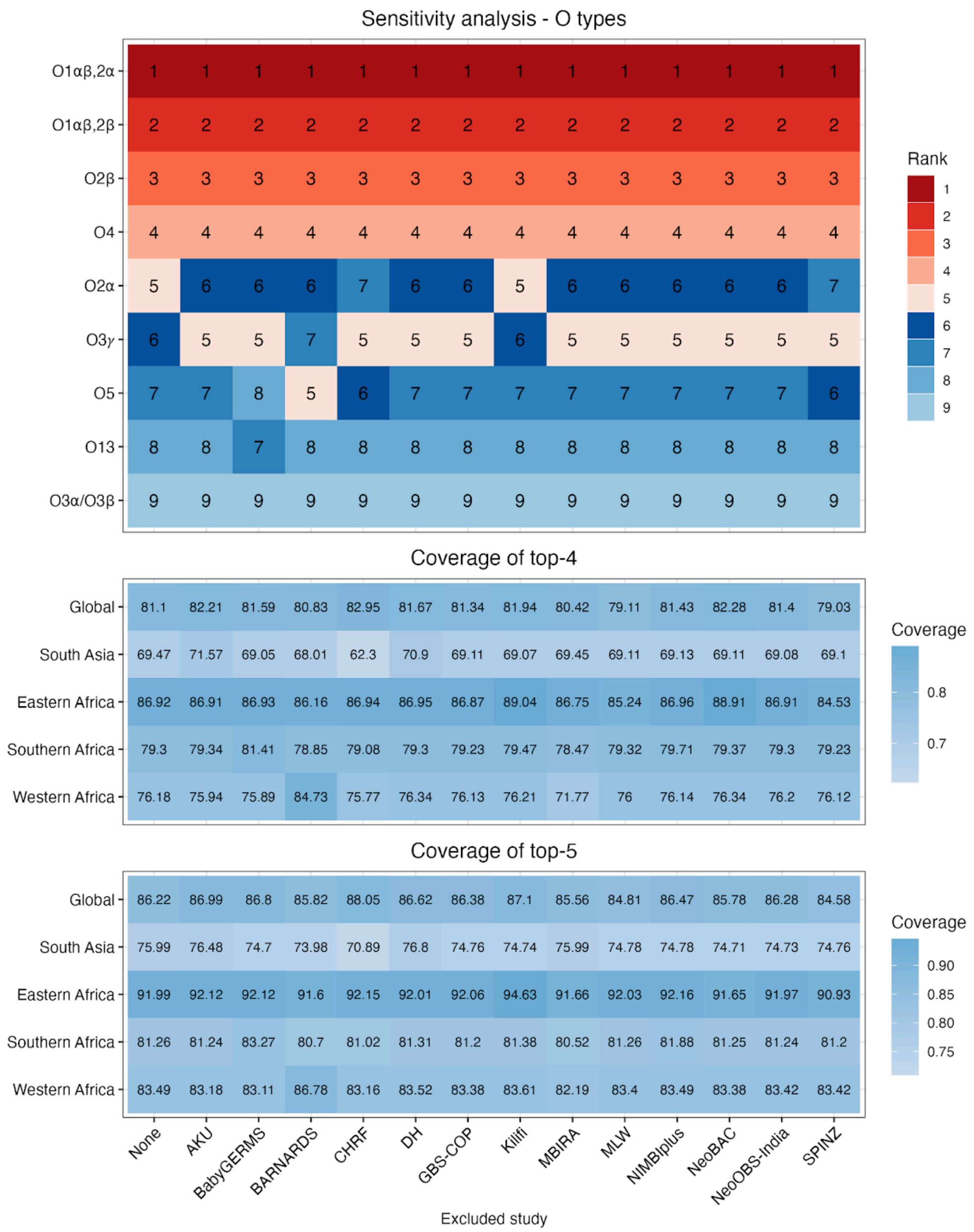

S2.3 Appendix: Effect of cluster adjustment on crude proportions per site.

Plots show crude proportion per site, estimated using raw (x-axis) vs cluster-adjusted (y-axis) counts, for the 4 most common K loci **(a-d)** and O types **(e-h)**. The impact of cluster-adjustment on the global prevalence estimates are shown in **S2.4 Appendix**.

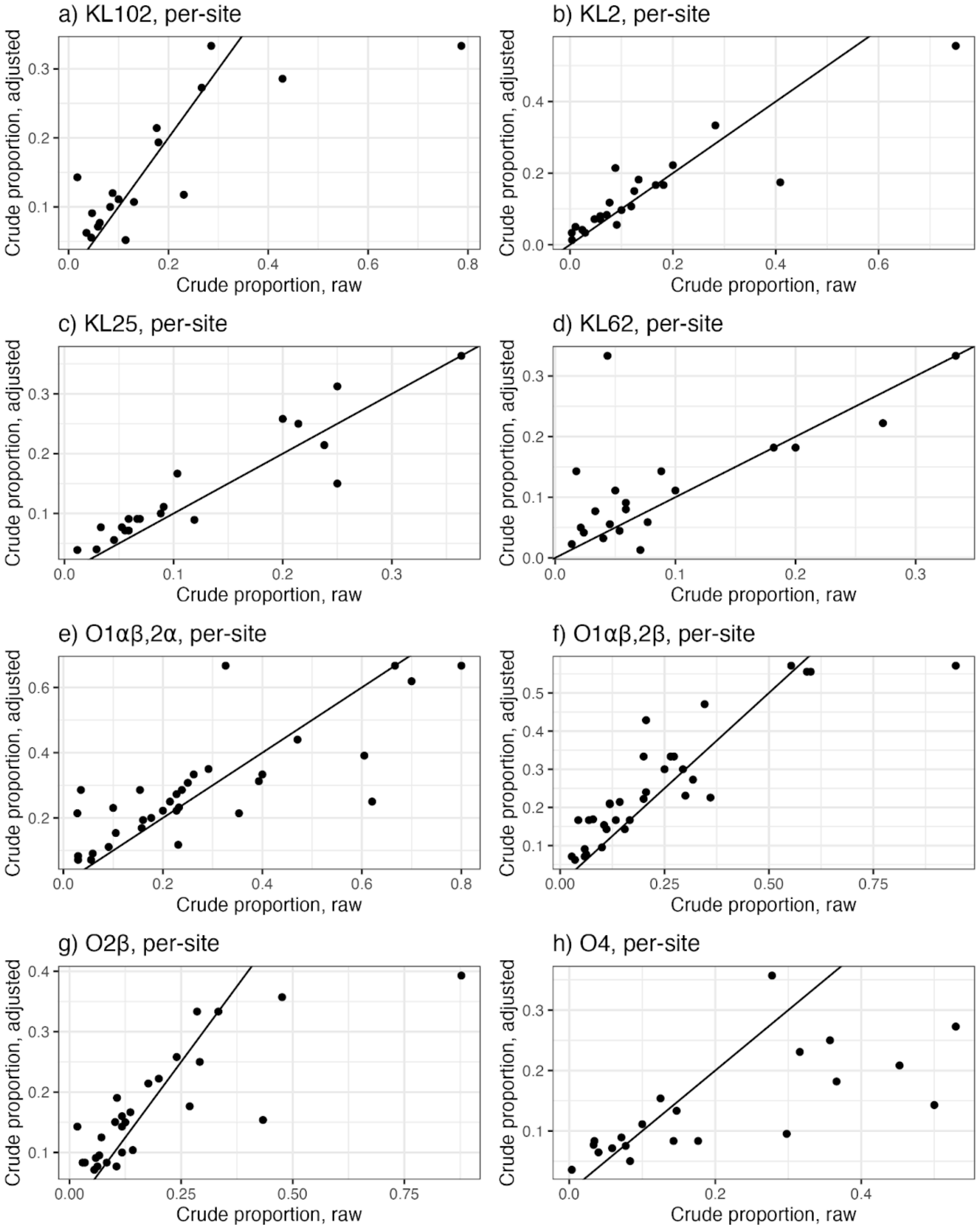

S2.4 Appendix: Effect of cluster adjustment on Bayesian modelled estimates of global K locus prevalence.

Global (overall) prevalence estimates modelled for **(a)** each K locus or **(b)** each predicted O type, using either raw counts (x-axis) vs cluster-adjusted counts (y-axis).

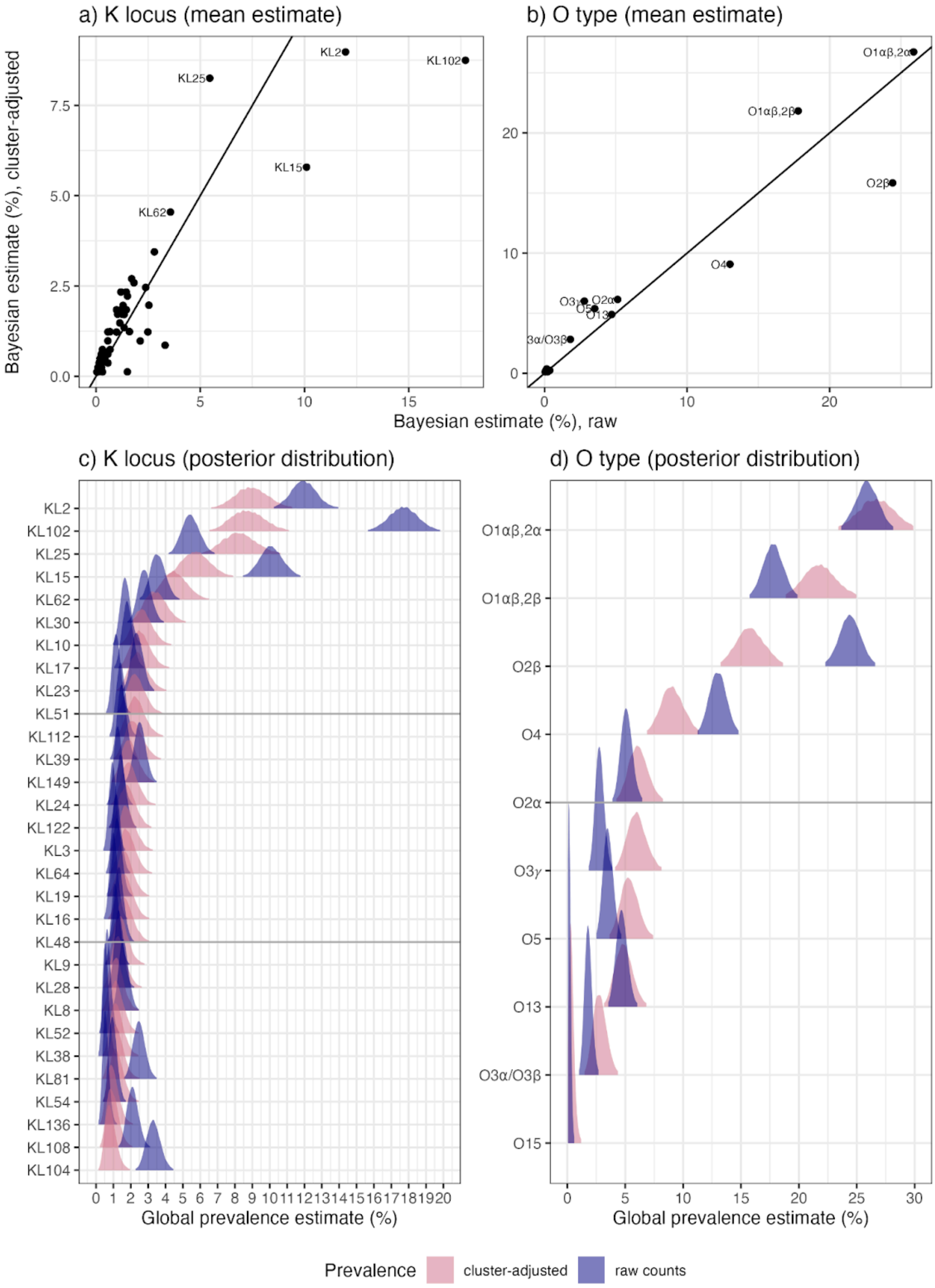

S2.5 Appendix: Sensitivity analysis showing effect of temporal clustering thresholds on K locus proportions and ranks (modelled global estimates).

The clustering threshold used for all primary analyses was 28 days; modelling was repeated using the 365-day threshold to assess sensitivity to clustering parameters. Data plotted here summarise the Bayesian modelled estimates for global prevalence, using counts adjusted for clustering using different temporal thresholds (≤28 days vs ≤365 days). In panels (**a-d**) each point represents a single K locus or O type. Panels (**e-f**) show the posterior distributions of prevalence estimates.

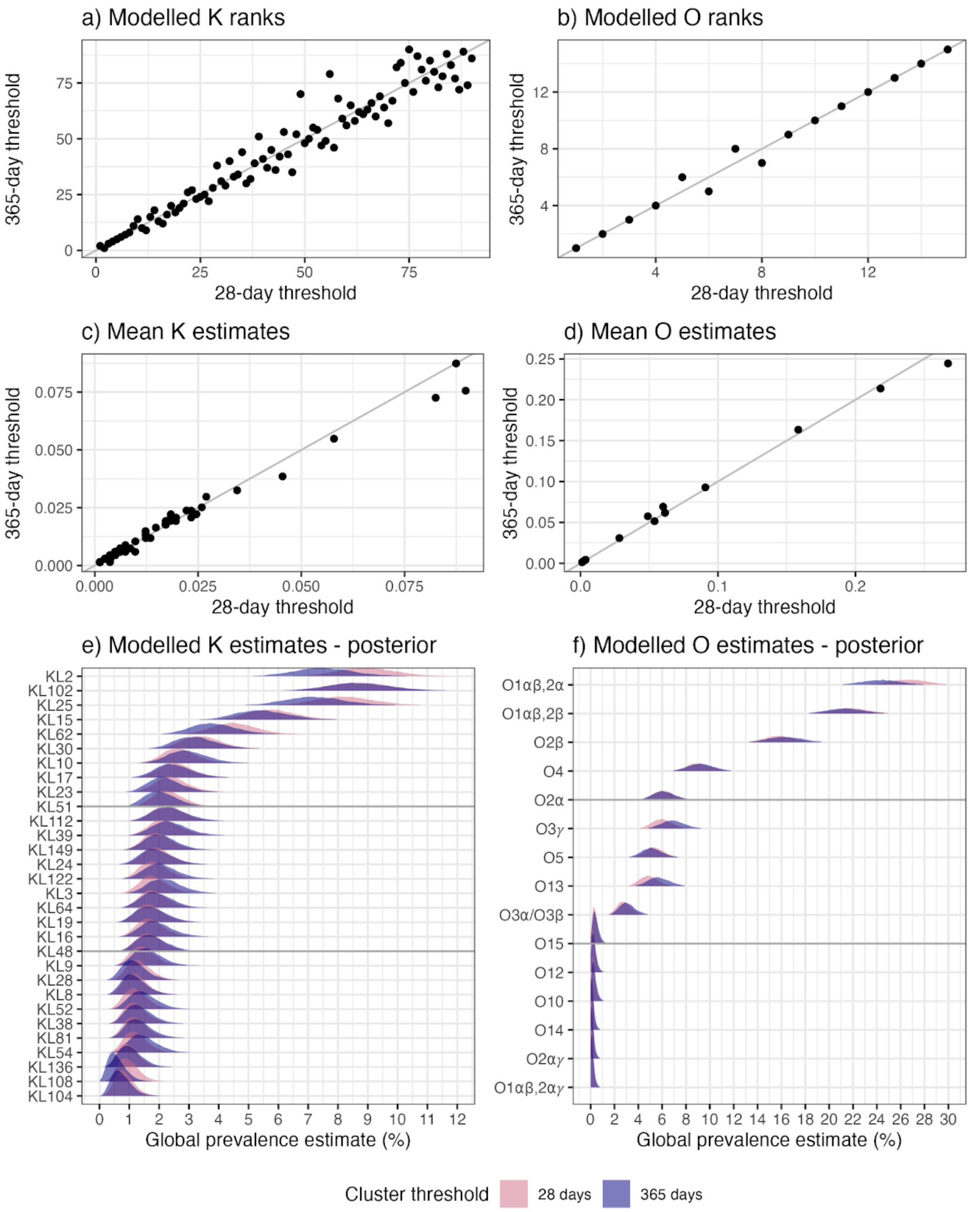

S2.6 Appendix: Sensitivity analysis showing effect of temporal clustering thresholds on K locus proportions and ranks (crude proportions).

Plotted data are based on crude proportions (rather than Bayesian modelled estimates of prevalence), using a SNP threshold of 10. The thresholds used for primary analysis were 28 days (red) and 10 SNPs.

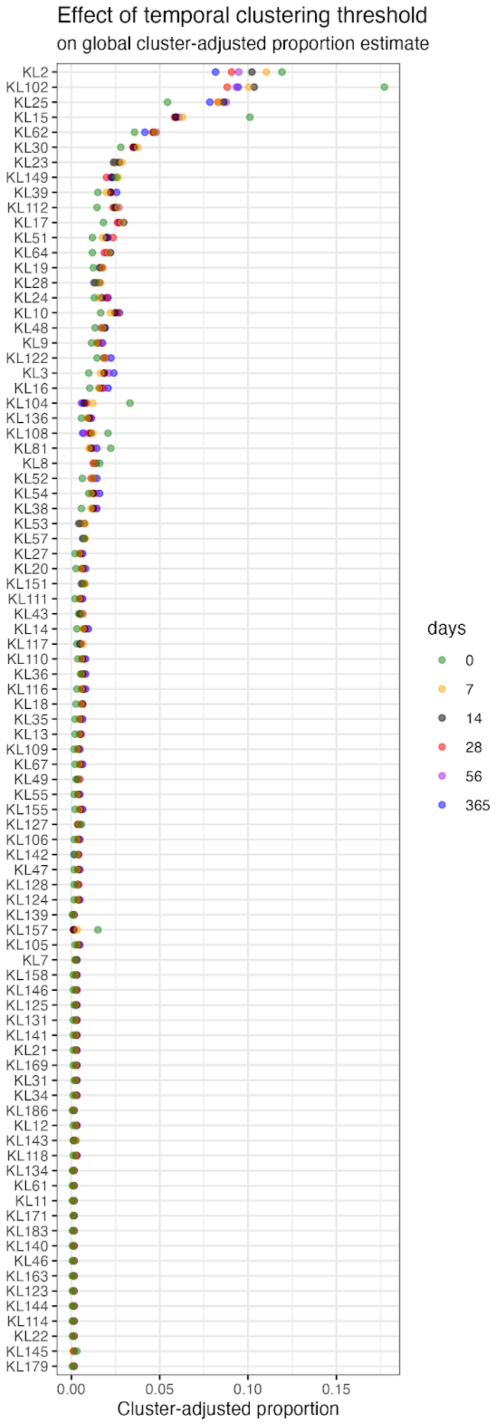

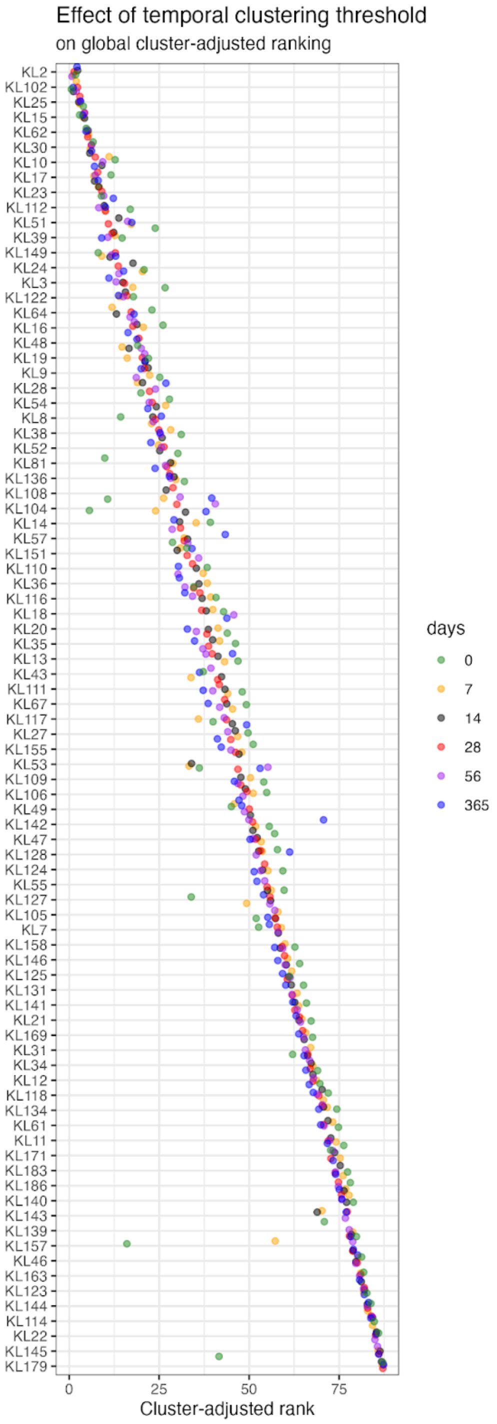

S2.7 Appendix: Sensitivity analysis showing effect of SNP clustering thresholds on K locus proportions and ranks (crude proportions).

Plotted data are based on crude proportions (rather than Bayesian modelled estimates of prevalence), using a temporal threshold of 4 weeks. The thresholds used for primary analysis were 10 SNPs (red) and 4 weeks.

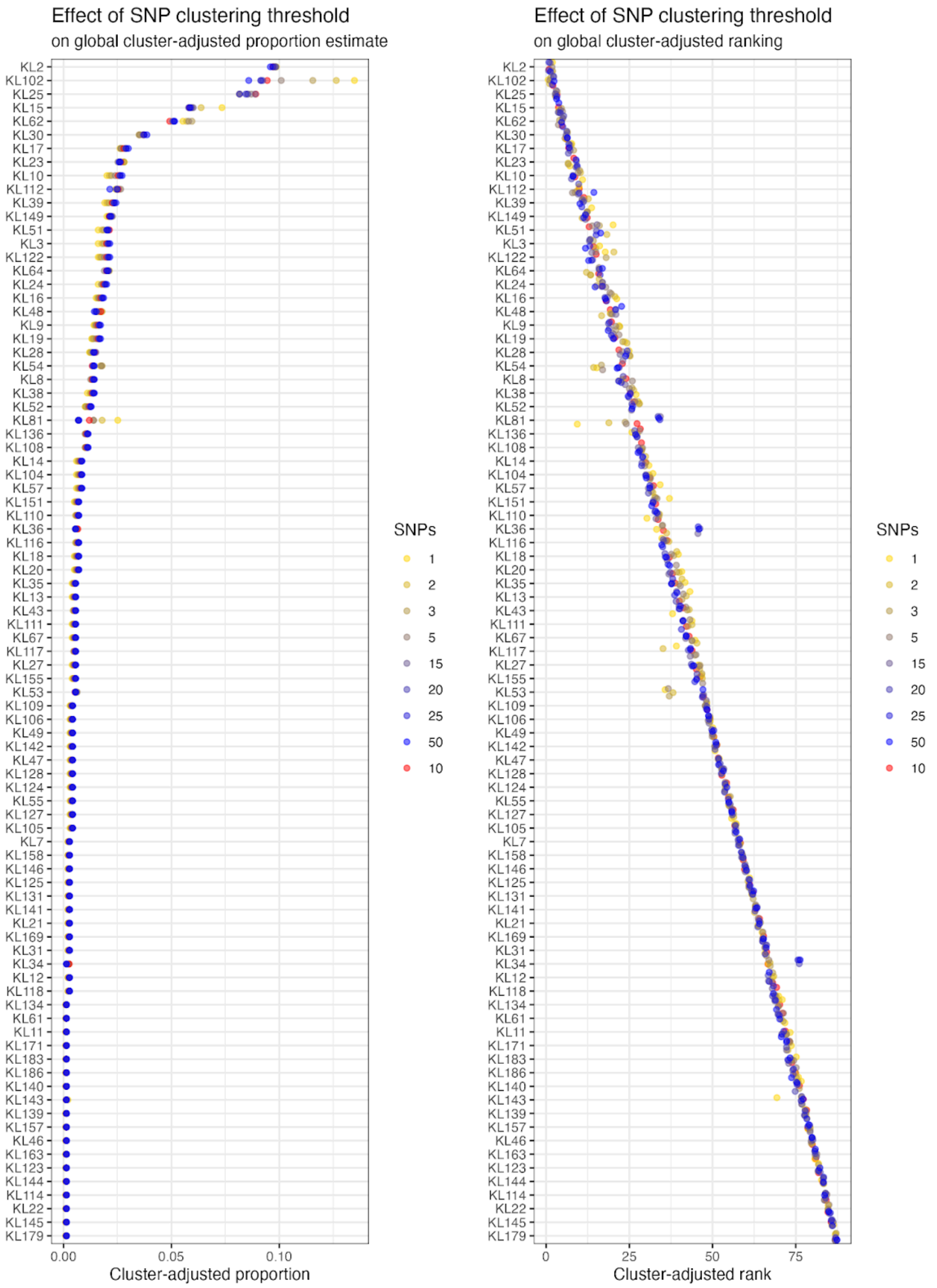

S2.8 Appendix: Coverage estimates for KL set 1 (global top 20)

Cumulative coverage estimates for the top 20 K loci (KL), ordered by cluster-adjusted global prevalence estimate. The first panel shows estimates for all cases (“All”), remaining panels show coverage estimated for the subset of cases indicated in the panel title (ESBL, extended-spectrum beta-lactamase; CP, carbapenemase-producing). Coverage estimated from raw counts are shown as solid lines, and from cluster-adjusted counts as dashed lines, each with 95% credible intervals shaded.

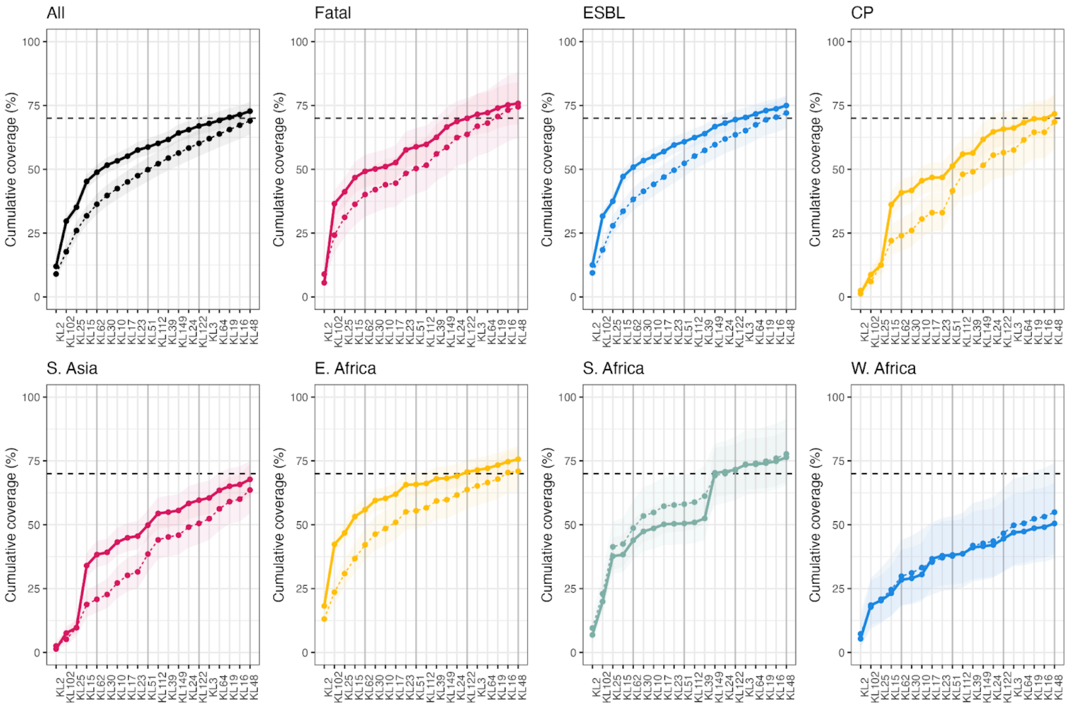

S2.9 Appendix: Coverage estimates for KL set 2 (top-8 per region)

Cumulative coverage estimates for KL set 2, comprising the top-8 K loci within each region, ranked according to cluster-adjusted global prevalence estimate. The first panel shows estimates for all cases (“All”), remaining panels show coverage estimated for the subset of cases indicated in the panel title (ESBL, extended-spectrum beta-lactamase; CP, carbapenemase-producing). Coverage estimated from raw counts are shown as solid lines, and from cluster-adjusted counts as dashed lines, each with 95% credible intervals shaded.

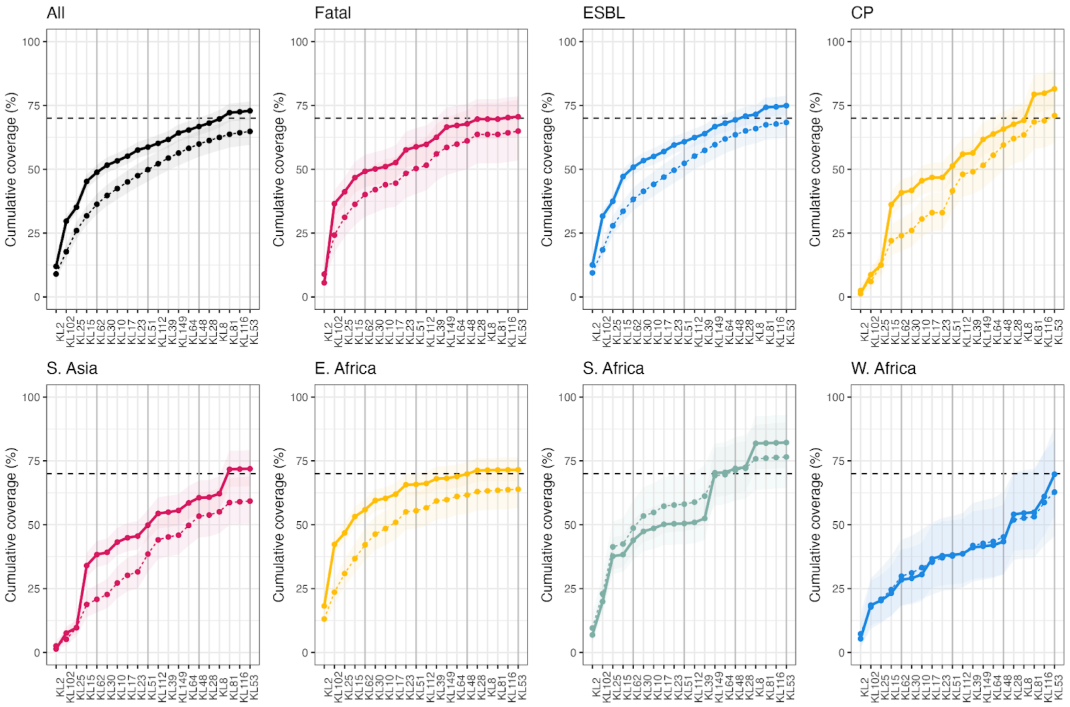

S2.10 Appendix: Coverage estimates for O types

Cumulative coverage estimates for the top 10 O types, ordered by cluster-adjusted global prevalence estimate. The first panel shows estimates for all cases (“All”), remaining panels show coverage estimated for the subset of cases indicated in the panel title (ESBL, extended-spectrum beta-lactamase; CP, carbapenemase-producing). Coverage estimated from raw counts are shown as solid lines, and from cluster-adjusted counts as dashed lines, each with 95% credible intervals shaded.

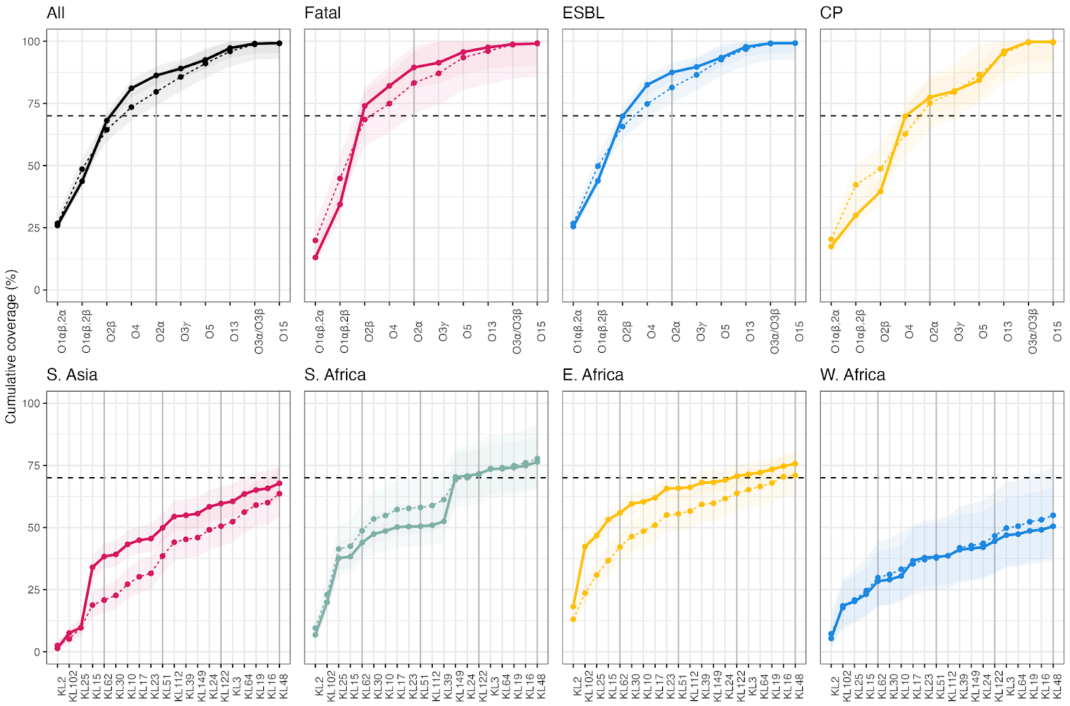

Supplement: S2 Appendix — (PDF) [file pmed.1004879.s013.pdf]
