# Supplementary material for: Distribution of capsule and O types in Klebsiella pneumoniae causing neonatal sepsis in Africa and South Asia: A meta-analysis of genome-predicted serotype prevalence to inform potential vaccine coverage
Source: PLoS Med. 2026 Jan 12;23(1):e1004879. doi: 10.1371/journal.pmed.1004879 (PMC12810917; doi:10.1371/journal.pmed.1004879)
Supplement: S3 Appendix — (PDF) [file pmed.1004879.s014.pdf]

S3 Appendix: Model Diagnostics.

We evaluated three key diagnostic metrics to ensure the robustness of our Bayesian inference: Rhat, the effective sample size ratio, and divergent transitions.

Rhat is a metric of convergence. It compares the between-chain variance to the within-chain variance, providing an indication of whether multiple Markov chains have converged to the same target distribution. Values close to 1 suggest that the chains have mixed well; values substantially above 1 may indicate potential issues with convergence. In our models, we checked that the maximum Rhat were under 1.01. All values were markedly below this (see table below).

The effective sample size ratio measures sampling efficiency by estimating the number of effectively independent samples obtained from the correlated MCMC draws. Since autocorrelation reduces the number of independent samples, a higher ratio indicates that the sampling process is efficient and that the posterior estimates are reliable. We computed the minimum effective sample size ratio for each model to confirm that our inference is based on a sufficiently large number of independent draws. All models, with the exception of models a and b, had minimum effective sample size ratios of 0.1 (see table below) or above indicating samples were sufficiently independent. These models were rerun with more iterations as described in the Methods. When re-run, those models which still had a ratio of less than 0.1 were then inspected closely to determine whether this was a feature of all effective sample size ratios, or rare aberrations. It was determined that these low ratios impacted very few (as low as 1) model parameters, and as such the models were deemed acceptable.

Divergent transitions occur when the sampler encounters regions in the posterior distribution with challenging geometry, which can lead to biased estimates. The absence of any divergent transitions in our diagnostics confirms that the sampler was able to explore the posterior landscape smoothly and without numerical instabilities.

Model diagnostics for all Bayesian models

Primary models

Model ID	subgroup	counts	days	antigen	max Rhat	min Neff_ratio
a	CP	adj	28	K	1.00088238	0.03796967
b	CP	raw	28	K	1.00140556	0.0548587
c	CP	adj	28	O	1.00202916	0.1828553
d	CP	raw	28	O	1.00121229	0.18043056
e	ESBL	adj	28	K	1.00180625	0.23567658
f	ESBL	raw	28	K	1.00178756	0.25413868
g	ESBL	adj	28	O	1.00171688	0.23119961
h	ESBL	raw	28	O	1.00202841	0.26851334
i	Fatal	adj	28	K	1.00203937	0.19809457
j	Fatal	raw	28	K	1.00277878	0.1854011
k	Fatal	adj	28	O	1.001967	0.2824846
l	Fatal	raw	28	O	1.00137218	0.24379735
m	Full	adj	28	K	1.00269449	0.2116118
n	Full	raw	28	K	1.00216508	0.24603945
o	Full	adj	28	O	1.00151937	0.29722619
p	Full	raw	28	O	1.00196789	0.27290005
q	Full	adj	365	K	1.00146703	0.246428
r	Full	raw	365	K	1.00189497	0.253698
s	Full	adj	365	O	1.00166388	0.29827219
t	Full	raw	365	O	1.00144951	0.26270787

Leave-one-out analysis

Model ID	study left out	type	days	antigen	max Rhat	min Neff_ratio
1	DH	adj	28	K	1.00201008	0.22653089
2	DH	raw	28	K	1.00162068	0.25393721
3	AKU	adj	28	K	1.00193839	0.22544558
4	AKU	raw	28	K	1.00244137	0.24700401
5	Baby GERMS-SA	adj	28	K	1.00124264	0.23064113
6	Baby GERMS-SA	raw	28	K	1.00255461	0.24534982
7	BARNARDS	adj	28	K	1.00167169	0.25459179
8	BARNARDS	raw	28	K	1.00177469	0.23131859
9	NIMBIplus	adj	28	K	1.00194693	0.18831693
10	NIMBIplus	raw	28	K	1.00154699	0.23196231

11	CHRF	adj	28	K	1.00221728	0.21047287
12	CHRF	raw	28	K	1.00224478	0.25348302
13	GBS-COP	adj	28	K	1.00338847	0.20016976
14	GBS-COP	raw	28	K	1.00167867	0.25835135
15	Kilifi	adj	28	K	1.00275235	0.20073946
16	Kilifi	raw	28	K	1.0017595	0.26507194
17	MBIRA	adj	28	K	1.00463533	0.16901181
18	MBIRA	raw	28	K	1.00304928	0.2466877
19	MLW	adj	28	K	1.00144484	0.21951426
20	MLW	raw	28	K	1.0015223	0.25765252
21	NeoBAC	adj	28	K	1.00188468	0.21835634
22	NeoBAC	raw	28	K	1.00205798	0.22931353
23	SPINZ	adj	28	K	1.00213139	0.2262595
24	SPINZ	raw	28	K	1.00169702	0.24546798
25	DH	adj	28	O	1.00203495	0.27384274
26	DH	raw	28	O	1.00106341	0.25978561
27	AKU	adj	28	O	1.00200069	0.26262671
28	AKU	raw	28	O	1.00148952	0.26859157
29	Baby GERMS-SA	adj	28	O	1.00128027	0.27731336
30	Baby GERMS-SA	raw	28	O	1.00166639	0.27880282
31	BARNARDS	adj	28	O	1.00236419	0.27353777
32	BARNARDS	raw	28	O	1.00178975	0.27661917
33	NIMBIplus	adj	28	O	1.00095957	0.28238137
34	NIMBIplus	raw	28	O	1.00121788	0.26769635
35	CHRF	adj	28	O	1.00135568	0.27264555
36	CHRF	raw	28	O	1.0015541	0.25169272
37	GBS-COP	adj	28	O	1.00139764	0.30402441
38	GBS-COP	raw	28	O	1.00199053	0.27497652
39	Kilifi	adj	28	O	1.00176647	0.26223239
40	Kilifi	raw	28	O	1.00099135	0.29196706
41	MBIRA	adj	28	O	1.00202812	0.29837529
42	MBIRA	raw	28	O	1.00199366	0.25439415
43	MLW	adj	28	O	1.00106562	0.28017804
44	MLW	raw	28	O	1.00133002	0.26457843
45	NeoBAC	adj	28	O	1.00119297	0.27130553
46	NeoBAC	raw	28	O	1.00144345	0.27021013
47	SPINZ	adj	28	O	1.00132456	0.28506661
48	SPINZ	raw	28	O	1.00175768	0.25543517
