# Supplementary material for: Distribution of capsule and O types in Klebsiella pneumoniae causing neonatal sepsis in Africa and South Asia: A meta-analysis of genome-predicted serotype prevalence to inform potential vaccine coverage
Source: PLoS Med. 2026 Jan 12;23(1):e1004879. doi: 10.1371/journal.pmed.1004879 (PMC12810917; doi:10.1371/journal.pmed.1004879)
Supplement: S1 Protocol — (PDF) [file pmed.1004879.s016.pdf]

Study Protocol

Genomic surveillance of Gram-negative bacteria causing neonatal sepsis

12 Jan 2024, v2

Sponsor: London School of Hygiene & Tropical Medicine
Funder: Bill & Melinda Gates Foundation
LSHTM Ethics reference: 29931

Table of Contents

Background **2**
- Purpose of project 3
- Overview of study procedures 3

Aims and objectives **4**
- Overall aim 4
- Objectives 4

Detailed study procedures **5**
- Inclusion criteria 5
- Data management 5
- Statistical analysis 6
- Outcome measures 9

Ethical approvals **10**

Research outputs **10**
- Reporting of results 10
- Data sharing 10

References **10**

Background

It is estimated nearly 1 million newborns each year die in the first month of life from sepsis [1-2]. The most common agents are Gram negative bacteria, and the most common species is *Klebsiella pneumoniae* [2-4]. *K. pneumoniae* are intrinsically resistant to ampicillin and readily acquire resistance to other drugs relied on for treatment [5]. This resistance, together with inadequate access to antibiotics in low- and middle-income countries (LMICs), contributes to high rates of morbidity and mortality associated with *K. pneumoniae* sepsis in these settings [6-7].

The source of infection is unclear in most cases. *K. pneumoniae* is a common coloniser of the gut and skin of humans, and the bacteria can be passed vertically from mother to baby [8]. Nosocomial transmission of *K. pneumoniae* can also occur, including in neonatal intensive care units in high- and low-income settings [9-11], via contaminated equipment, reagents, plumbing, and other sources. Infection prevention and control (IPC) measures in neonatal care units is therefore likely be important for disease prevention. However the relative contribution to neonatal sepsis disease burden of nosocomial transmission (in the hospital), vs vertical transmission (from the mother), remains unclear particularly in LMIC settings.

Another potential prevention strategy is maternal immunisation against *K. pneumoniae,* which could theoretically protect newborns via transplacental antibody transfer. It was recently estimated [12] that a vaccine effective against 70% of *K. pneumoniae* strains, with coverage coverage equivalent to current effective maternal tetanus vaccination coverage levels [13], could avert ~400,000 neonatal sepsis cases and ~80,000 neonatal deaths annually worldwide. The largest benefits would be in Africa and Asian countries where up to 6% of all neonatal deaths could theoretically be averted [12].

There are not yet any licensed vaccines against *K. pneumoniae*, however those in development and early trials target the capsular polysaccharide (K) and/or lipopolysaccharide (O) antigens [14]. *K. pneumoniae* can express at least 80 different K antigens and 13 different O antigens, therefore any vaccine targeting these polysaccharides would need to be multi-valent, aiming to induce immunity against multiple K and/or O types in order to protect against a majority of infections. There is no theoretical maximum for the number of polysaccharide antigens that can be included in a single vaccine, however there is a licensed 23-valent vaccine against *Streptococcus pneumoniae* (targeting 23 K types), and a 24-valent vaccine against 24 *K. pneumoniae* K types was found to be safe and immunogenic in a small trial of ten adult trauma patients [15]. It is therefore assumed that, in principle, a conjugate vaccine targeting up to 25 *K. pneumoniae* K and/or O polysaccharide antigens could be feasible.

The distribution of antigens amongst *K. pneumoniae* sepsis cases in LMICs is not yet known. Serotyping of *K. pneumoniae* is complex and very rarely performed, and so understanding the diversity and distribution of these antigens relies on predictions from bacterial whole genome sequence (WGS) data [16]. A study of isolates from adult bloodstream infections in seven Asian countries estimated that the top 20 K antigens would provide coverage of 60-75% of cases in

each country [16]. Another study of infecting and colonizing isolates in Malawi showed a similar rate (75% coverage by the top 20 K antigens) [17]. These studies show that the same or better coverage could theoretically be achieved by a vaccine effective against a small number of O antigens (n=2-5), however there is evidence that O antigen vaccines against *K. pneumoniae* may not be reliably immunogenic, as the presence of capsule interferes with the binding of anti-O antibodies [18].

Purpose of project

The overall purpose of this project is to utilise pathogen sequence data to inform efforts to prevent neonatal sepsis in LMICs in Africa and Asia, using maternal vaccination and hospital IPC interventions. This will be done by aggregating bacterial WGS data from neonatal sepsis cases captured in multiple studies in African and Asian countries, and conducting meta-analyses to (i) estimate global and regional prevalence of K and O antigens; (ii) identify combinations of antigen targets that could maximise the number of neonatal sepsis cases protected against; and (iii) estimate the proportional burden of neonatal sepsis cases that are associated with nosocomial transmission.

The purpose of conducting these analyses is to (i) inform the development of vaccines against neonatal sepsis; and (ii) to provide evidence for the degree to which nosocomial transmission contributes to disease burden, in order to motivate and justify investments in IPC interventions and provide a baseline against which to evaluate their effectiveness.

The focus will be neonatal sepsis caused by *K. pneumoniae*, as it is the most common single agent and is associated with high rates of antimicrobial resistance and mortality. However *Escherichia coli* and other species will also be analysed as far as the available data allows. The scope of the study is all neonatal sepsis cases, however subgroup analyses will also be conducted to estimate vaccine coverage and transmission burden specifically for (i) fatal cases, and (ii) drug-resistant cases (extended-spectrum beta-lactamase [ESBL] producing and carbapenemase-producing [CP]).

Overview of study procedures

WGS data will be sourced from studies focused on neonatal sepsis (surveillance and/or intervention trials) or cause-of-death in LMICs in Africa and Asia. Data will be filtered such that each case is represented by a single bacterial genome (representing the infecting agent), and only neonates (up to 30 days old) are included. WGS data will be analysed using a consistent set of free open-source bioinformatics tools developed by the KlebNET Genomic Surveillance Platform (GSP). Data owners (i.e. principal investigators of the primary studies) will be asked to run these tools on their WGS data, and extract key results fields (K and O antigen predictions; presence of extended-spectrum beta-lactamases and carbapenemases; multi-locus sequence types (STs); pairwise genetic distances) to contribute to this project. This minimises the need to share raw unpublished WGS data that is not needed for the current study, while ensuring a consistent bioinformatics approach to primary sequence analysis. Limited patient data will also be provided by the study PIs, as required to meet the meta-analysis aims (age in days, or

confirming that all cases were ≤30 days old; study site [i.e. hospital]; date of specimen collection from which the bacterial isolate was grown; outcome of sepsis episode [at 30-day follow-up, coded as died, discharged, or still in hospital]).

The data will be analysed in order to estimate the prevalence of each K and O antigen, per study site, per country, per region, and globally. Prevalence data will be treated as simple proportions of cases. For meta-analysis, data will be combined across studies to estimate antigen prevalence (per country, region, globally), by (i) pooling numerators and denominators to generate simple weighted estimates, and (ii) by using Bayesian meta-analysis to generate pooled estimates and 95% credible intervals for individual antigens and combinations of antigens. These prevalence estimates will be used to estimate the theoretical coverage of neonatal sepsis cases by sets of the most common antigens (e.g. top-5, top-10, up to top-25); and also the coverage of subgroups (fatal cases and drug-resistant cases).

Data from neonatal sepsis studies (not cause-of-death studies) will be used to estimate transmission burden. This will be done by assigning cases to clusters on the basis of temporal distance (days) and genetic distance between consecutive cases, using single-linkage clustering. Sensitivity analyses will be conducted to identify the most appropriate primary threshold for temporal and genetic distance, and to estimate the proportional transmission burden (and 95% confidence interval) across a range of thresholds.

Aims and objectives

Overall aim

The overall aim of the project is to undertake meta-analyses of pathogen sequence data from multiple studies to inform efforts to prevent neonatal sepsis in LMICs in Africa and Asia, specifically (1) maternal vaccination and (2) hospital IPC interventions.

The focus will be neonatal sepsis caused by *K. pneumoniae*, as it is the most common single agent and is associated with high rates of antimicrobial resistance and mortality. However *Escherichia coli* and other species will also be analysed as far as the available data allows.

Objectives

Specific objectives are to (for each pathogen):

1. Estimate global and regional prevalence of K and O antigens.
2. Identify combinations of antigen targets that could maximise the number of neonatal sepsis cases protected against; and
3. Estimate the proportional burden of neonatal sepsis cases that are associated with nosocomial transmission.

The scope of the study is all neonatal sepsis cases, however subgroup analyses will also be conducted under each objective to estimate vaccine coverage and transmission burden specifically for (i) fatal cases, and (ii) drug-resistant (ESBL and CP) cases.

Detailed study procedures

Inclusion criteria

Studies will be included in the meta-analysis if they meet the following criteria:
• Study site/s located in a LMIC of Asia or Africa.
• Study protocol includes recruitment of paediatric sepsis cases, with bacterial culture confirmation of causative agents; OR
• Study protocol involves post-mortem assessment of child deaths, with blood culture of causative agents to which deaths from infection were attributed.
• Bacterial blood culture isolates from cases were subjected to WGS (as part of original study protocol, or as an add-on).
• For subgroup analysis of fatal cases: study recruited paediatric sepsis cases, AND recorded death/discharge at 30 days post-recruitment

Cases within these studies will be included if they meet the following inclusion criteria:
• Neonate, aged up to 30 days post birth at the time of recruitment / specimen collection
• Blood culture positive for *K. pneumoniae* (or pathogen of interest) and WGS available for the cultured isolate
• WGS data meets the minimal sequence quality criteria established by KlebNET-GSP (<500 contigs, genome size 5-6.2 Mbp)
• Date of specimen collection available
• For subgroup analysis of fatal cases: participant has death/discharge data available for 30 days post-recruitment

Individual study sites will be included in meta-analyses if (i) the **study** meets the inclusion criteria; AND (ii) the study site includes **at least 10 cases** that meet the inclusion criteria.

Data management

Anonymised patient and genome-derived data will be received from individual study PIs in tabular formats and collated and processed using R.

The data will include one row per participant, including:

1) Participant information:
    - anonymised participant identifiers
    - age in days, or confirming that all cases were ≤30 days old;
    - study site (i.e. hospital);
    - date of specimen collection from which the bacterial isolate was grown;
    - outcome of sepsis episode

    (at 30-day follow-up, coded as died, discharged, or still in hospital; where available)
2) Pathogen genome information, extracted from KlebNET-GSP analysis outputs:

- K and O antigen predictions (generated using Kaptive v2 [16])
- presence of extended-spectrum beta-lactamases and carbapenemases (generated using Kleborate v2 [19])
- multi-locus sequence types (STs) (generated using Kleborate v2 [19])
- pairwise genetic distances to all other cases from the same study site (generated using Pathogenwatch [20])

The data will be subjected to validation and standardisation (e.g. imposing uniform capitalisation and age formats, fixing spelling errors, coding missing data appropriately, etc) and combined (using R) into a single database for analysis. Incomplete or conflicting data identified during this data validation will be queried with the principal investigators of the relevant primary study, who will be asked to check/confirm/complete data accordingly.

For published studies, the principal investigators of the primary study will be:

- asked to agree for their data to be included in the study;
- asked to confirm the data available from their primary study, fill in gaps, and correct errors;
- asked to provide details of consent and ethical approvals relevant to the provided data;
- given the opportunity to contribute to interpretation and reporting of the results as co-authors.

For unpublished studies, principal investigators of primary studies who are contributing data to this meta-analysis project will be:

- asked to provide data from their primary study, fill in gaps, and correct errors;
- asked to confirm their agreement for the provided data to be included in the study;
- asked to provide details of consent and ethical approvals relevant to the provided data;
- given the opportunity to contribute to interpretation and reporting of the results as individually named co-authors.

Statistical analysis

Adjusting for clustering. Published genomic analysis of neonatal sepsis cases [3; 21] shows strong clustering at some sites, which reflects nosocomial transmission of *K. pneumoniae*. These highly-localised outbreaks could lead to overestimation of the prevalence of the K/O loci associated with the outbreak strain, resulting in serotype prevalence estimates that are highly biased by chance events that are specific to the study site and sampling period. To adjust for the effect of localized outbreaks, we will identify putative transmission clusters as outlined below. We will consider each unique cluster at a given study site as resulting from a single index case, and estimate an adjusted prevalence of each locus type (details below) using these index cases only (i.e. ignoring any secondary cases resulting from nosocomial spread). The resulting adjusted prevalences are expected to better reflect the distribution of serotypes in the circulating pathogen populations that serve as a source of neonatal sepsis infections, without the influence of chance outbreaks. To ensure a simple and reproducible approach to cluster identification, we

will use pairwise SNP distances calculated by the KlebNET-GSP Pathogenwatch platform (PW distances). These distances are calculated across a *K. pneumoniae* core gene library (1,972 genes, 2,172,367 bp) [20]. There is emerging consensus across multiple studies that a threshold of 21-25 genome-wide SNPs (estimated using the more common method of mapping reads to a *K. pneumoniae* reference genome) is suitable for identifying nosocomial transmission clusters of *K. pneumoniae* [22-24]. We recently showed that a genome-wide threshold of 25 SNPs is equivalent to a PW distance of 10 SNPs in *K. pneumoniae* [25], hence we will use a threshold of ≤10 PW SNPs and ≤365 days, between genomes isolated from the same study site, to define putative nosocomial transmission clusters for our primary analysis.

Prevalence $p(i,s)$ of each antigen, i, at each site s, will be calculated as a simple proportion:

$$p(i,s) = x(i,s) / n(s);$$

where $x(i,s)$ is the number of infections with antigen i at site s, and $n(s)$ is the total number of infections observed at site s.

Confidence intervals (95%) for these proportions were calculated as:

$$p(i,s) \pm 1.96 * \text{sqrt}\left(p(i,s) * [1-p(i,s)] / n(s)\right).$$

Cluster-adjusted prevalence $p_{adj}(i,s)$ of each antigen, i, at each site s, will be calculated in the same manner but using the counts of unique clusters (of size n=1 or more infections) instead of infection counts:

$$p_{adj}(i,s) = x_{\text{clusters}}(i,s) / n_{\text{clusters}}(s);$$

where $x_{\text{clusters}}(i,s)$ is the number of putative clusters of infections with antigen i at site s, and $n_{\text{clusters}}(s)$ is the total number of putative clusters of infections observed at site s, and clusters were defined as described above.

Weighted means of raw or cluster-adjusted antigen prevalence will be estimated at different geographical levels (country, region, global) by summing the numerators and denominators in the above equations. Prevalence $p(i,y)$ of antigen i in country or region y, was calculated as:

$$p(i,y) = \sum_{s=1}^{N} x(i,s) / \sum_{s=1}^{N} n(s) \text{ for all } N \text{ sites } (s) \text{ in country } y;$$

and cluster-adjusted prevalence $p_{adj}(i,y)$ of each antigen, i, in country or region y, as:

$$p_{adj}(i,y) = \sum_{s=1}^{N} x_{\text{clusters}}(i,s) / \sum_{s=1}^{N} n_{\text{clusters}}(s) \text{ for all N sites (s) in country y.}$$

This is equivalent to a weighted mean prevalence per country, using sample size as weights. (Note while inverse-variance weights are generally preferred, they are not suitable here as zero counts are common in the data and this creates infinite weights. Zero counts are expected in data of this kind, as the number of antigens exceeds the sample size at most sites and so only a subset of antigens have non-zero counts at any given site.)

Pooled global estimates of raw or cluster-adjusted antigen prevalence will be obtained using Bayesian random-effects models fit to count data using the R package brms [26].

To estimate global prevalence of each antigen, $p(i)$, we will model the individual-level count data ($x(i)$ and n) per study site, and assume these observations are random draws from a binomial

distribution. We will use an intercept-only model, and interpret the intercept as the population effect size (global prevalence of antigen *i*) that we wish to estimate (logit-transformed). We will include a random-effects term (1 | site) as we assume each study site has a different true effect size, which represent random draws from an overarching population of true effect sizes. The model specification in brms will therefore be:

x(i) | trials (*n*) ~ 1 + (1 | site).

Two global models will be fit per antigen, i, by running 4 chains with 20,000 iterations each using (i) raw and (ii) cluster-adjusted counts.

Cumulative global prevalence: As the prevalence of each antigen is estimated separately, they are not constrained to sum to zero. Therefore to estimate the cumulative global prevalence of a set of antigens *k={i, j,...}* of size K, we will calculate the sum of the individual component antigen counts at each site as follows:

$k(i,s) = \sum_{i=1}^{K} x(i,s)$

and fit a separate model for each set, *k*:

k(i) | trials (*n*) ~ 1 + (1 | site).

For each model, the posterior distribution of the pooled population effect size will be transformed using the inverse-logit, $exp^{posterior} / (1+ e^{posterior})$, and summarised to obtain the median value and 2.5% and 97.5% quantiles, which we interpret as the point estimate and 95% credible interval for the global prevalence *p(i, global)* or *p(k, global)*.

Pooled regional estimates of antigen *i* prevalence per region (or country) *y*, *p(i, y)*, will be estimated using a similar approach to the global model but including a random-effects term for region (as we assume each region has a different true distribution of effect sizes, but not a fixed effect size), and forcing an intercept per region (by including a zero term). The model specification in brms will be:

x(i) | n ~ 0 + (1 | region) + (1 | site).

Cumulative regional prevalence of sets of antigens will be estimated as above, by substituting the combined total for all antigens *i* in set *k,* i.e. *k(i),* for *x(i)* in the model. For each model, the posterior distributions of effect sizes for each region intercept will be transformed using the inverse-logit and summarised as above, to obtain point estimates and 95% credible intervals for prevalence in each region *y*, i.e. *p(i, y)* or *p(k, y)*.

Sensitivity analysis for prevalence estimates will be undertaken using a leave-one-out approach, to explore the extent to which estimates are influenced by any single study. The above models will be re-run once per study, excluding that study, and global and regional prevalence estimated as above. The focus of this analysis will be to determine whether the composition of the top 5, 10, 15, 20 and 25 loci is altered by the inclusion/exclusion of any given study, or if the cumulative coverage estimates of the globally ranked top 5, 10, 15, 20, 25 loci are significantly impacted by any one study.

Subgroup analyses will be undertaken to estimate the prevalence of K and O antigens, and the coverage of the global top-5/10/15/20/25 antigens, amongst i) fatal cases; ii) ESBL cases; iii) CP cases. This will be achieved by filtering out cases not belonging to the subgroup of interest, and re-calculating the antigen prevalence and cumulative coverage estimates using the methods outlined above.

Transmission burden per site will be estimated by using single-linkage clustering to identify putative transmission clusters (with thresholds of ≤10 PW SNPs and ≤28 days), and quantifying (i) the number and proportion of cases in clusters; and (ii) the number and proportion of cases attributable to transmission, assuming each cluster includes one index case plus secondary cases, i.e. number attributable = number of cases in clusters minus the number of clusters. The 95% confidence intervals for each proportion estimate will be calculated using the standard formula (as given above for prevalence proportions). Sensitivity analysis will be conducted using SNP thresholds of n=1 to 20 and temporal thresholds of 1 to 52 weeks, to explore how proportional burden estimates change with thresholds.

Outcome measures

For Objective 1, the primary outcome measure will be the Bayesian estimate of global cluster-adjusted prevalence per antigen, with 95% credible intervals. Secondary measures will include Bayesian estimates of regional and national cluster-adjusted prevalence; simple weighted estimates of global, regional and national cluster-adjusted prevalence; and subgroup estimates (i.e. global, regional and national cluster-adjusted prevalence of antigens amongst fatal, ESBL and CP cases).

For Objective 2, the primary outcome measures will be the cumulative coverage of the top 5, 10, 15, 20, or 25 antigens (ranked according to the Bayesian estimate of global cluster-adjusted prevalence per antigen, in Objective 1), estimated using Bayesian modelling and with 95% credible intervals, for all neonatal sepsis cases. Secondary measures will include cumulative coverage estimates per region and country, and per subgroup (fatal, ESBL and CP cases).

For Objective 3, the primary outcome measure will be the proportion of neonatal sepsis cases estimated to be attributable to nosocomial transmission (with 95% confidence intervals), per site, using the optimal thresholds for temporal and genetic distances. Secondary outcome measures will be the proportion of neonatal sepsis cases involved in transmission clusters, and the proportion of cases involved in transmission clusters or attributable to transmission, across a range of thresholds.

Ethical approvals

Ethical approval will be sought from the LSHTM HREC to include each dataset in this project's meta-analysis. The principal investigator for each such study will be asked to confirm that the appropriate local approvals are in place for the primary study including use of the study data in secondary research analyses, and to provide details of participant consent and ethical approvals for the primary study (including HREC details and consent forms where relevant).

Research outputs

Reporting of results

Results of these meta-analyses will be reported in the form of scientific papers, one on antigen distributions and one on transmission burden. The manuscripts will be posted as preprints on completion, and then submitted to open-access academic journals for peer review. PIs of the primary studies will be invited to contribute to the interpretation and presentation of results in these publications, and to be included as co-authors. Research outputs will indicate the primary source publications for each included dataset, with corresponding references provided for each primary study where available.

Data sharing

All WGS-derived data and anonymised clinical or source information used in this study will be made public, as supplementary data accompanying the manuscripts (both on submission to a preprint server, and when published in final peer-reviewed form).

References

1. Liu, L., et al., Global, regional, and national causes of child mortality in 2000–13, with projections to inform post-2015 priorities: an updated systematic analysis. Lancet, 2015. 385(9966):430–440.
2. Okomo, U., et al., Aetiology of invasive bacterial infection and antimicrobial resistance in neonates in sub-Saharan Africa: a systematic review and meta-analysis in line with the STROBE-NI reporting guidelines. Lancet Infect Dis, 2019. 19(11): p. 1219-1234.
3. Sands, K., et al., Characterization of antimicrobial-resistant Gram-negative bacteria that cause neonatal sepsis in seven low- and middle-income countries. Nat Microbiol, 2021. 6(4): p. 512-523.
4. Hamer, D.H., et al., Etiology of bacteremia in young infants in six countries. Pediatr Infect Dis J, 2015. 34(1): p. E1-8.
5. Wyres, K.L. and Holt, K.E. *Klebsiella pneumoniae* as a key trafficker of drug resistance genes from environmental to clinically important bacteria. Curr Opin Microbiol, 2018. 45:131-139

6. Chaurasia S., et al., Neonatal sepsis in South Asia: huge burden and spiralling antimicrobial resistance. BMJ, 2019. 22;364:k5314.
7. Thomson, K.M., et al., Effects of antibiotic resistance, drug target attainment, bacterial pathogenicity and virulence, and antibiotic access and affordability on outcomes in neonatal sepsis: an international microbiology and drug evaluation prospective substudy (BARNARDS). Lancet Infect Dis, 2021. 21(12):1677–1688.
8. Okomo, U., et al., Maternal colonization and early-onset neonatal bacterial sepsis in the Gambia, West Africa: a genomic analysis of vertical transmission. Clin Microbiol Infect, 2023. 29(3):386.e1-386.e9.
9. Okomo, U., et al., Investigation of sequential outbreaks of *Burkholderia cepacia* and multidrug-resistant extended spectrum β-lactamase producing *Klebsiella* species in a West African tertiary hospital neonatal unit: a retrospective genomic analysis. Lancet Microbe, 2020. 1(3):e119-e129.
10. Chung The, H., et al., A high-resolution genomic analysis of multidrug-resistant hospital outbreaks of *Klebsiella pneumoniae*. EMBO Mol Med, 2015. 7(3):227-39.
11. Robinson, M.L., et al., Maternal Colonization Versus Nosocomial Transmission as the Source of Drug-Resistant Bloodstream Infection in an Indian Neonatal Intensive Care Unit: A Prospective Cohort Study. Clin Infect Dis, 2023. 5;77(Suppl 1):S38-S45.
12. Kumar C.K., et al., Global, regional, and national estimates of the impact of a maternal Klebsiella pneumoniae vaccine: A Bayesian modeling analysis. PLoS Med, 2023. 20(5): e1004239.
13. Vandelaer, J., et al., Tetanus in developing countries: an update on the Maternal and Neonatal Tetanus Elimination Initiative. Vaccine, 2003. 21(24):3442–3445.
14. Choi, M., et al., Progress towards the development of Klebsiella vaccines. Expert Rev Vaccines, 2019. 18(7):681–691.
15. Campbell WN, Hendrix E, Cryz S Jr, Cross AS. Immunogenicity of a 24-valent Klebsiella capsular polysaccharide vaccine and an eight-valent Pseudomonas O-polysaccharide conjugate vaccine administered to victims of acute trauma. Clin Infect Dis, 1996. 23(1):179–181.
16. Lam, M.M.C., et al., Kaptive 2.0: updated capsule and lipopolysaccharide locus typing for the *Klebsiella pneumoniae* species complex. Microb Genom, 2022. 8(3):000800.
17. Lewis, J.M., et al., Genomic and antigenic diversity of colonizing *Klebsiella pneumoniae* isolates mirrors that of invasive isolates in Blantyre, Malawi. Microb Genom, 2022. 8(3):000778.
18. Wantuch, P.L., et al., Capsular polysaccharide inhibits vaccine-induced O-antigen antibody binding and function across both classical and hypervirulent K2:O1 strains of *Klebsiella pneumoniae*. PLoS Pathog, 2023. 19(5):e1011367.
19. Lam, M.M.C., et al., A genomic surveillance framework and genotyping tool for Klebsiella pneumoniae and its related species complex. Nat Comms, 2021. 12:4188.
20. Argímon, S., et al., Rapid Genomic Characterization and Global Surveillance of Klebsiella Using Pathogenwatch. Clin Infect Dis, 2021. 73(Suppl_4):S325-S335.
21. Heinz, E., et al., Longitudinal analysis within one hospital in sub-Saharan Africa over 20 years reveals repeated replacements of dominant clones of *Klebsiella pneumoniae* and

stresses the importance to include temporal patterns for vaccine design considerations. MedRxiv Preprint Server, 2023. DOI: 10.1101/2023.09.26.23296137.
22. Sherry, N.L., et al., Genomics for Molecular Epidemiology and Detecting Transmission of Carbapenemase-Producing Enterobacterales in Victoria, Australia, 2012 to 2016. J Clin Microbiol, 2019. 57(9):e00573-19.
23. David, S., et al., Epidemic of carbapenem-resistant Klebsiella pneumoniae in Europe is driven by nosocomial spread. Nat Microbiol, 2019. 4:1919.
24. Gorrie, C.L., et al., Gastrointestinal Carriage Is a Major Reservoir of Klebsiella pneumoniae Infection in Intensive Care Patients. Clin Infect Dis, 2017. 65(2):208-215.
25. Foster-Nyarko, E., et al., Nanopore-only assemblies for genomic surveillance of the global priority drug-resistant pathogen, *Klebsiella pneumoniae*. Microb Genom, 2023. 9(2):mgen000936.
26. Bürkner, P., Advanced Bayesian Multilevel Modeling with the R Package brms. The R Journal, 2018. 10(1), 395-411.

Project	69b Contact providing data and approval details	69a(i) Purpose and methods	34c Consent details	69 Approvals
**BabyGERMS**	Nelesh Govender, University of the Witwatersrand and National Institute for Communicable Diseases, South Africa	Population-level surveillance for bloodstream infections and meningitis among neonates aged <28 days in South Africa. Tier 1 includes national surveillance of culture-confirmed neonatal infections at all public-sector hospitals describing infection incidence risk, pathogen profile and antimicrobial susceptibility by institution, province and healthcare level (2014-2021). Tier 2 (nested within tier 1) conducted at six regional neonatal units over 12 months, comparing clinical characteristics of neonates with early-onset and late-onset infections and aiming to identify potentially modifiable risk factors for mortality. Tier 2 includes assessing antimicrobial susceptibility of neonatal pathogens, evaluating the appropriateness of empiric antibiotic prescribing, and investigating genomic epidemiology pathogens (via WGS). DOI: 10.1136/bmjopen-2021-049070	Clinical data were collected retrospectively from medical records. A waiver of consent was obtained from the HREC for use of this data, and associated bacterial isolates cultured as part of routine diagnostics, for the surveillance research.	The study has been approved by the Human Research Ethics Committee of the University of the Witwatersrand (M190320). Approvals for the tier 2 surveillance study were received from each provincial research committee through registration on the National Health Research Database.
**KWTRP surveillance**	Anne Amulele, KEMRI-Wellcome Trust Research Programme, Kenya	Hospital-based surveillance for bloodstream infections in paediatric admissions, including pathogen etiology (causative agent) and outcome, at Kilifi County Hospital (KCH), Kiambu sub-County Hospital and Mbagathi County Hospital. This nested study uses this material to address *Klebsiella pneumoniae* specifically, characterising isolates via WGS to investigate the K and O serotypes responsible for neonatal sepsis, and assessing anti-*Klebsiella* antibodies in blood samples from mothers and babies.	Written informed consent was obtained from the child's mother or legal guardian, and covers sharing of de-identified data and samples with other researchers.<br><br>**See attached consent form.**	The use of surveillance study materials for the nested *K. pneumoniae* study was approved by the KEMRI Scientific and Ethics Review Unit, ref 281/4687, on 17/4/2023. A research license was obtained from the National Commission for Science, Technology and Innovation, ref 527823, license no. NACOSTI/P/23/26005, on 31/5/2023.

**GBS COPS**	Shabir Madhi, University of the Witwatersrand, South Africa	The primary objective was to assess correlates of protection in neonates against invasive infection with Group B *Streptococcus*. Surveillance was conducted for bloodstream infections in neonates, including pathogen etiology, patient serology (presence of antibodies against Group B *Streptococcus*), and outcome of sepsis. *Klebsiella pneumoniae* was found to be the most common causative agent of sepsis, and isolates were further characterised using WGS to explore serotype variation.	Written informed consent was obtained from the child's mother or legal guardian, and covers sharing of de-identified data and samples with other researchers, including data on organisms other than Group B *Streptococcus*.<br><br>**See attached consent form.**	University of the Witwatersrand, Human Research Ethics Committee (HREC), ref 181110, 24/4/2023.
**MLW Biobank**	Nicholas Feasey, MLW	Queen Elizabeth Central Hospital (QECH) in Blantyre, Malawi has undertaken sentinel surveillance of bacteraemia and meningitis in partnership with the Malawi-Liverpool Wellcome Programme (MLW) since 1998. All isolates phenotypically identified as *K. pneumoniae* were sequenced, irrespective of resistance profile or patient demographic, from 1996 to 2020, with no change in sampling strategy from 2000 - 2020.<br>This study aimed to investigate the shifts in resistance profile and population structure of the bacterial population over time, and highlight the need to consider trends over time and not just total numbers for considerations of new drug regimens or vaccine target selection. It also aimed to improve understanding of transmission within the hospital to help focus IPC interventions.	Surveillance data were collected retrospectively from medical records. A waiver of consent was obtained from the HREC for use of this data, and associated bacterial isolates cultured as part of routine diagnostics, for the research.	Ethical approval for this study was granted by the University of Malawi College of Medicine Research Ethics Committee (COMREC) (P.11/18/2541) and isolates were shipped under a Nagoya Protocol compliant Access and Benefit Sharing agreement between the Government of Malawi and the Wellcome Sanger Institute.
**SPINZ**	David Hamer, Boston University, USA	Prospective cohort study in a neonatal intensive care unit in Zambia, before and after implementing an infection prevention bundle. Assessed incidence and mortality of neonatal sepsis, particularly for hospital associated infections (onset after 3 days in hospital), and effectiveness of the infection prevention bundle on these outcomes (trial number NCT02386592). Blood	Written informed consent was obtained from the neonate's mother or legal guardian in English or the most common local languages, Nyanja and Bemba. This covers	Ethical approval was granted by the Boston University Medical Center Institutional Review Board, USA (ref H-33473), 21/3/2016; and the Excellence in Research

		culture was used to identify pathogens and investigate prevalence of nosocomial bacteremia caused by drug-resistant Gram negative bacterial infections. WGS was conducted subsequently to investigate drug resistance mechanisms and transmission.<br>DOI: 10.1093/cid/ciy1114.	additional analysis of laboratory and medical record data after completion of the primary study.<br><br>**See attached consent form.**	Ethics and Science (ERES) CONVERGE, Zambia (ref 2015-Jan-004), 1/3/2015.
**CHAMPS**	Dianne Blau, US CDC; Shabir Madhi, University of the Witwatersrand, South Africa	CHAMPS seeks to identify definitive causes of stillbirths and child deaths. CHAMPS surveillance sites utilize notification systems which report all under-5 deaths and stillbirths to the local team within 24 hours of the child's death. Eligible cases are enrolled and the cause of death investigated through postmortem minimally invasive tissue sampling (MITS), laboratory investigations, and caregiver interviews; followed by expert panel review to assign a final cause of death. Sites are in seven countries: Baliakandi, Bangladesh; Harar and Kersa, Ethiopia; Siaya and Kisumu, Kenya; Bamako, Mali; Manhiça, Mozambique; Bombali, Sierra Leone; and Soweto, South Africa.<br><br>*Klebsiella pneumoniae* was identified in the causal chain of 21% of child deaths for which cause of death was assigned (DOI: 10.1016/s2666-5247(23)00290-2). Isolates were sequenced to characterise virulence factors, drug resistance and K/O antigens. Bacterial genome data, together with country and date of isolation of the bacteria, and age group of the child from which it was isolated, (0-30 days, 31-60 days, 61-365 days, 1-5 years) will be provided for inclusion in the meta-analysis.<br><br>Project manual:<br>https://champshealth.org/resources/champs-manual/	Parents or guardians of deceased children provided written informed consent before collection of data, specimens, or information on the mothers.<br><br>Consent was requested specifically for use of deidentified data and biospecimens for downstream research.<br><br>A copy of the generic consent form that all sites use is enclosed. Each site translates into their own language and may make slight adaptations, but the language concerning the secondary use of specimens and data are not adapted.<br><br>**See attached consent form template.**	Ethical approval for the overall study and site-specific protocols was granted by the Emory University Institutional Review Board (ref 00091706). Ethics committees overseeing investigators at each also approved overall and site-specific protocols.

**Gambia**	Grant Mackenzie, MRC Gambia	Surveillance for meningitis, pneumonia and bloodstream bacterial infections in children was established in 2008 for the primary purpose of estimating the burden of *Streptococcus pneumoniae* infection, to inform the development and deployment of vaccines. In 2018, a vaccine trial was commenced and surveillance f continued under its protocol. *Klebsiella pneumoniae* were frequently isolated from blood samples throughout both studies, and stored for future analysis. WGS is now being conducted on all stored *K. pneumoniae* in order to estimate antigen prevalence and investigate nosocomial transmission.	Written informed consent was obtained from the child's parent or guardian, and covers storage of samples for future tests and research relating to infection.<br><br>**See attached consent form.**	The original surveillance study was approved by the Gambia Government/MRC Joint Ethics Committee (ref SCC 1087), 17/8/2007.<br>The vaccine trial was subsequently approved by the same committee (ref SCC 1577v1.2), 22/2/2018.
**NeoOBS**	Mike Sharland, St George's University London; Sally Ellis, GARDP	The primary objective of NeoOBS was to assess mortality rates of hospitalised infants being treated with significant sepsis. Secondary objectives included describing clinical presentation and recovery, sepsis management and microbiological epidemiology. This involved the collection of routine clinical, laboratory and antimicrobial therapy data from approximately 3000 neonates less than 60 days old with clinically diagnosed sepsis. Patients were followed for 28 days. Data from the study will be used to inform the design of future antibiotic treatment trials for neonatal sepsis. Nineteen study sites, from 11 countries in Asia (Bangladesh, China, India, Thailand, and Vietnam), Africa (Kenya, South Africa, and Uganda), Europe (Italy and Greece), and South America (Brazil)<br>Clinical trials register: NCT03721302.<br>DOI: 10.1371/journal.pmed.1004179	Written informed consent was obtained from parents or legal guardians for neonates to be enrolled in the study.<br><br>**See attached PIL and ICF from each site.**	Ethical approval was obtained from St. George's, University of London (SGUL) Research Ethics Committee and sites' local, central or national ethics committees as follows:<br>• SGUL REC, ref 2018.0153, 25/9/2018.<br>• Bangladesh: Bangladesh Institute of Child Health Ethical Review Committee, ref 01-04-2018, 16/9/2018.<br>• Brazil: Hospital das Clinicas da Faculdade de Medicina de Ribeirao, ref 08753619.0.1001.5440, 31/7/2019; Santa Casa de Misericordia de Sao, ref 08753619.0.2001.5479, 19/8/2019.<br>• China: Beijing Children Hospital, ref 2018-168, 26/09/2018; Beijing

				Obstetrics and Gynecology Hospital, ref 2018-KY-065-02, 17/12/2018; Shenzhen Children's Hospital Neonatology, ref 2018023, 18/10/2018.<br>• Greece: Hippokration Hospital, Thessaloniki, 21/11/2018.<br>• India: JIPMER, ref JIP/IEC/2018/061, 24/05/2018; Lady Hardinge Medical College, ref LHMC/ECHR/2018/56, 05/09/2018; King Edward Memorial Hospital and Seth Gordhandas Sunderdas Medical College, ref IEC (I)/OUZ/4245/2018, 12/12/2018.<br>• Italy: Ospedale Pediatrico Bambino Gesù, ref 1623/2018, 12/09/2023.<br>• Kenya: KEMRI, Kilifi County Hospital, ref KEMRI/RES/7/3/1, 07/11/2018.<br>• South Africa: Charlotte Maxeke Academic Hospital, Johannesburg, ref M180563, 25/05/2018; Chris Hani Baragwanath Academic Hospital, Johannesburg, ref M180539, 25/05/2018; Adrie Bekker and Angela

				Dramowski, ref 6982, 07/08/2018.<br>• Thailand: Chiang Rai Prachanukroh Hospital,ref CR 0032.102/10129, 12/06/2018; Queen Sirikit National Institute of Child Health, ref REC.099/2561, 06/08/2018.<br>• Uganda: Mu-Jhu Hospital, Kampala, ref REC REF 2019-012, 15/01/2019.<br>• Vietnam: Vietnam National Children's Hospital, ref VNCH-RICH-18-11, 10/08/2018.
**NIMBi**	Susan Coffin, Children's Hospital of Philadelphia	Prospective surveillance study aiming to investigate the epidemiology and microbial etiology of sepsis in neonates in Gaborone, Botswana. Protocol includes blood culture to identify agents of sepsis, and microbiome profiling of gut, skin and respiratory tract.	Written informed consent was obtained from the child's parent or guardian, and covers storage of samples for future infection related research and bacterial characterisation.<br><br>**See attached consent form.**	Approval was granted by the University of Pennsylvania IRB (ref 833786), 22/4/2020; the Children's Hospital of Philadelphia Research Institute IRB (ref 19-016848), 25/7/2020; the IRBs of Princess Marina Hospital IRB (PMH 2/11AII(372)), 20/3/2024; and the Health Research Development Committee (HRDC) in Botswana (ref HPRD 6/14/1), 15/2/2024.
**SHARE**	Corrado Cancedda, University of Botswana, Susan Coffin, Children's	Research project at Princess Marina Hospital, Gabarone aiming to enhance laboratory capacity to detect emerging antimicrobial resistance; address public health surveillance questions including surveillance for colonization with multi-drug resistant organisms (MDRO) as an infection prevention measure;	A waiver of informed consent was granted, including for future research (see Penn IRB Approval).	Approval was granted by the University of Pennsylvania IRB (ref 851492), 9/6/2020; the Princess Marina Hospital (ref PMH 2/2A(7)/201), 20/5/2022; the University of

	Hospital of Philadelphia	and building a national network to support infection prevention and control. Study includes storage of clinical isolates and MDRO screening of inpatients in the neonatal unit, with isolates characterised by WGS.		Botswana IRB (ref UBR/RES/IRB/BIO/205), 11/5/2022; and the Health Research & Development Committee (HRDC) in Botswana (HPDME:13/18/1), 21/4/2022.
**MBIRA**	Alex Aiken, LSHTM	Observational parallel cohort study of patients with bacteraemia caused by Enterobacterales (cases) and matched uninfected controls, carried out in 8 African hospitals. The primary aim was to quantify the association between drug resistance and hospital mortality rate among inpatients. Secondary objectives included exploring association between drug resistance and other clinical outcomes, subgroup analysis by site, age-group, bacterial species, and community vs hospital acquired infections. Bacteria isolated during the study were stored for subsequent microbiological characterisation, and isolates from neonates have been sequenced.	Written informed consent was obtained from the child's parent or guardian, and covers storage of samples for future infection related research and bacterial characterisation.<br><br>Consent forms for each study site were recently submitted to LSHTM HREC under ref 21236-4.	Approval was granted by the LSHTM HREC, ref 21236-4 (latest amendment approved 27 March 2024). That application includes details of all local HREC approvals and copies of consent forms.
**BARNARDS**	Tim Walsh and Kirsty Sands, University of Oxford, UK	Observational cohort study analysing sepsis rates and causative agents in neonates and infants aged 0–60 days in 12 hospitals in Bangladesh, Ethiopia, India, Nigeria, Pakistan, Rwanda, South Africa. Blood cultures were taken to identify causative pathogens (with cultured isolates characterised by WGS and antimicrobial susceptibility testing). Substudies addressed source of infection (vertical transmission from mother vs hospital acquired), and effects of various factors (antibiotic resistance, bacterial factors, antibiotic access) on outcome.<br>DOIs: 10.1038/s41564-021-00870-7, 10.1016/S1473-3099(21)00050-5	All mothers provided informed consent (forms written in local languages) and could withdraw from the study at any time.<br><br>**See attached consent form.**	Approval was granted by:<br>• Ethical Review Committee, Bangladesh Institute of Child Health, BICH-ERC-4/3/2015, 15/09/2015<br>• Boston Children's Hospital, IRB-P00023058, 11/08/2016<br>• Institutional Ethics Committee, National Institute of Cholera and Enteric Diseases and Institute of Post Graduate Medical Education and Research, A-I/2016-IEC, 17/11/2016

				• IPGME&R Research Oversight Committee, Inst/IEC/2016/508, 04/11/2016<br>• Kano State Hospitals Management Board, 8/10/1437AH, 13/07/2016<br>• Health Research Ethics Committee (HREC), National Hospital, Abuja, NHA/EC/017/2015, 27/04/2015<br>• Republic of Rwanda, National Ethics Committee, No342/RNEC/2015, 10/11/2015<br>• Stellenbosch University and Tygerberg Hospital, Research projects, Western Cape Government, N15/07/063 04/12/2015 and 02/02/2016
**AIIMS**	Dr Ramesh Agarwal, Dr Kajal Jain	Observational cohort study of bacterial pathogens causing sepsis in newborns, in seven district hospitals in India. Blood samples were taken from participants and cultured to identify and characterize the causative bacterial agents. Cultured pathogens were characterized by whole genome sequencing to investigate the lineage, antimicrobial resistance, K and O serotypes responsible for neonatal sepsis.	Written informed consent was obtained from parents or legal guardians for neonates to be enrolled in the study.<br><br>**See attached consent form.**	Approval was granted by the Institute Ethics Committee All India Institute of Medical Sciences, New Delhi; ref IEC-683/07.12.2018, RP-12/2018; on 18/12/2018
**AKU**	Dr Imran Nisar	Referral-based surveillance study of bacterial pathogens causing sepsis in newborns in hospitals in Pakistan. Gram-negative bacterial isolates cultured from the blood of newborns diagnosed with sepsis, as part of routine care, were sent to the Aga Khan	Verbal consent and participant information was obtained by telephone call, from parents or legal	Approval was granted by the Aga Khan University Ethics Review Committee; ref 2023-8485-25083, on 23/5/2023.

		University for characterization including whole genome sequencing. Primary aims were to understand the etiology, antimicrobial resistance, and antigen distribution of K. *pneumoniae* and other Gram-negative bacterial pathogens causing sepsis in newborns across Pakistan. Additional clinical and personal data was also obtained by recruiting and interviewing parents of children from whom blood culture isolates were obtained (this data is not used in the meta-analysis).	guardians for neonates to be enrolled in the study.<br><br>**See attached telephone script for obtaining and recording verbal consent.**	
**CHRF**	Samir Saha and Senjuti Saha, Child Health Research Foundation Bangaldesh	CHRF conducts microbiological diagnostics for paediatric patients at Dhaka Shishu Hospital, Bangladesh, and has ethical approval to store all cultured bacterial isolates in a biobank for future research. In 2016 they commenced sequencing of isolates from neonatal sepsis, and all blood culture isolates of *Klebsiella pneumoniae* stored between 2004 and 2021 have now been characterised via whole genome sequencing to investigate the lineage, antimicrobial resistance, K and O serotypes responsible for paediatric sepsis amongst Bangaldeshi children. No personal or clinical data is included.	Written informed consent was obtained from parents or legal guardians for neonates to have their bacterial isolates included in the biobank for future research.<br><br>**See attached consent form.**	Approval was granted by the Bangladesh Institute of Child Health HREC, last updated to include biobanking of isolates from a broader range of infections, ref BCH-ERC-02-02-2021 on 3/2/2021.

London School of Hygiene & Tropical Medicine
Keppel Street, London WC1E 7HT
United Kingdom
Switchboard: +44 (0)20 7636 8636

www.lshtm.ac.uk

Observational / Interventions Research Ethics Committee

Professor Kathryn Holt

LSHTM

15 January 2024

Dear Kathryn

Study Title: Genomic surveillance of Gram-negative bacteria causing neonatal sepsis

LSHTM Ethics Ref: 29931

Thank you for responding to the Observational Committee's request for further information on the above research and submitting revised documentation.

The further information has been considered on behalf of the Committee by the Chair.

Confirmation of ethical opinion

On behalf of the Committee, I am pleased to confirm a favourable ethical opinion for the above research on the basis described in the application form, protocol and supporting documentation as revised, subject to the conditions specified below.

Conditions of the favourable opinion

Approval is dependent on local ethical approval having been received, where relevant.

Approved documents

The final list of documents reviewed and approved by the Committee is as follows:

Document Type	File Name	Date	Version
Consent form	GBS_CoP_ICF	01/08/2019	3
Consent form	SCC 1433 ICF Paeds v4.5	18/03/2021	4.5
Other	Holt Research_Ethics_online_training_certificate	28/07/2021	1
Investigator CV	HoltKE_CV_Aug2023	28/11/2023	1
Local Approval	Table v1 - Local Approvals	13/12/2023	1
Protocol / Proposal	Study Protocol v1	13/12/2023	1
Covering Letter	CoverLetter_Response_240112	12/01/2024	1
Protocol / Proposal	Study Protocol v2	12/01/2024	2

After ethical review

The Chief Investigator (CI) or delegate is responsible for informing the ethics committee of any subsequent changes to the application. These must be submitted to the Committee for review using an Amendment form. Amendments must not be initiated before receipt of written favourable opinion from the committee.

The CI or delegate is also required to notify the ethics committee of any protocol violations and/or Suspected Unexpected Serious Adverse Reactions (SUSARs) which occur during the project by submitting a Serious Adverse Event form.

An annual report should be submitted to the committee using an Annual Report form on the anniversary of the approval of the study during the lifetime of the study.

At the end of the study, the CI or delegate must notify the committee using an End of Study form.

All aforementioned forms are available on the ethics online applications website and can only be submitted to the committee via the website at: http://leo.lshtm.ac.uk

Additional information is available at: www.lshtm.ac.uk/ethics

Yours sincerely,

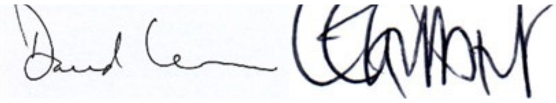

Professor David Leon and Professor Clare Gilbert
Co-Chairs

ethics@lshtm.ac.uk
http://www.lshtm.ac.uk/ethics/

London School of Hygiene & Tropical Medicine
Keppel Street, London WC1E 7HT
United Kingdom
Switchboard: +44 (0)20 7636 8636

www.lshtm.ac.uk

Research Ethics Committee

Professor Kathryn Holt

LSHTM

6 June 2024

Dear Kathryn,

Study Title: Genomic surveillance of Gram-negative bacteria causing neonatal sepsis

LSHTM Ethics ref: 29931 - 01

Thank you for submitting your amendment for the above research project.

Your amendment has been assessed by the Research Governance & Integrity Office and has been approved as a non-substantial change. The amendment does not require further ethical approval from the *observational* ethics committee.

List of documents reviewed:

Document Type	File Name	Date	Version
Local Approval	Gambia_Approval_17Aug07	17/08/2007	1
Local Approval	CHAMPS Consent Template	29/08/2013	1.3
Local Approval	SPINZ_ERES_Approval	11/03/2015	1
Local Approval	SPINZ_Consent_18May2015	18/05/2015	1.1
Local Approval	SPINZ_BUMC_Renewal_Approval	04/06/2015	1
Local Approval	SPINZ_BUMC_Renewal	21/03/2016	1
Local Approval	14. NeoOBS - India - PIS V1.0 JIPMER - English	02/01/2018	1.0
Local Approval	Gambia_Approval_22Feb18	22/02/2018	1
Local Approval	12. NeoOBS - India - Consent V2.0 JIPMER - English	23/05/2018	2.0
Local Approval	11. NeoOBS - India - JIPMER Institutional Ethical Committee (human research) Certificate	24/05/2018	1
Local Approval	NeoOBS -South Africa- m180539 ethics clearance certificate	25/05/2018	1
Local Approval	NeoOBS -South Africa-EC Approval letter- Charlote Maxeke	25/05/2018	1
Local Approval	NeoOBS -Thailand- Chiang Rai -_EC Approval Letter_PHPT_EN	12/06/2018	1
Local Approval	16. NeoOBS - India - Ethics clearence from AIIMS	06/07/2018	1
Local Approval	10. NeoOBS - CSC Decision letter_KEMRI, Kilifi County Hosptial	06/07/2018	1
Local Approval	NeoOBS__Bangladesh_ PIL 12.07.18_V2.0	12/07/2018	2.0
Local Approval	NeoOBS__Bangladesh_ICF Final 12.07.18_V3.0_Dhaka Shishu	12/07/2018	3.0
Local Approval	12. NeoOBS ICF 12.07.18_V3.0_wih DoW	12/07/2018	2.0
Local Approval	13. NeoOBS ICF 12.07.18_V3.0	12/07/2018	3.0
Local Approval	12. NeoOBS_PIL and ICF_Italia_v2.2_20180712	12/07/2018	2.2
Local Approval	9. NeoOBS -South Africa- ICF Final 12.07.18_V3.0_Baragwanath	12/07/2018	3.0
Local Approval	10. NeoOBS -South Africa- ICF Final 12.07.18_V3.0_wih DoW_Baragwanath	12/07/2018	3.0
Local Approval	11. NeoOBS -South Africa- PIL Final 12.07.18_V2.0_Baragwanath	12/07/2018	2.0
Local Approval	9. NeoOBS -South Africa- ICF Final 12.07.18_V3.0_Charlotte Maxeke	12/07/2018	3.0
Local Approval	10. NeoOBS -South Africa- ICF Final 12.07.18_V3.0_wih DoW_Charlotte Maxeke	12/07/2018	3.0

Document Type	File Name	Date	Version
Local Approval	11. NeoOBS -South Africa- PIL Final 12.07.18_V2.1_Charlotte Maxeke	12/07/2018	2.1
Local Approval	9. NeoOBS -Thailand- Chiang Rai - Master Version ICF Final 12.07.18_V3.0	12/07/2018	3.0
Local Approval	10. NeoOBS -Thailand- Chiang Rai - ICF Master Version Final 12.07.18_V3.0_wih DoW	12/07/2018	3.0
Local Approval	11. NeoOBS -Thailand- Chiang Rai - PIL Master Version FINAL 12.07.18_V2.0	12/07/2018	2.0
Local Approval	9. NeoOBS -Thailand- QSNIC- ICF 12.07.18_V3.0	12/07/2018	3.0
Local Approval	10. NeoOBS-Thailand- QS ICF 12.07.18_V3.0_wih DoW	12/07/2018	3.0
Local Approval	11. NeoOBS-Thailan PIL Master Version FINAL 12.07.18_V2.0	12/07/2018	2.0
Local Approval	9. NeoOBS - Vietnam - PIL 12.07.18_V2.0_English	12/07/2018	2.0
Local Approval	10. NeoOBS - Vietnam - Parents Information Leaflet (Vietnamese) (Ver 2.0)	12/07/2018	2.0
Local Approval	11. NeoOBS - Vietnam - ICF 12.07.18_V3.0_english	12/07/2018	3.0
Local Approval	12. NeoOBS - Vietnam - ICF 12.07.18_V3.0_wih DoW	12/07/2018	3.0
Local Approval	13. NeoOBS - Vietnam - Consent form (Vietnamese) (version 3.0)	12/07/2018	3.0
Local Approval	NeoOBS -South Africa-Tygerberg NeoOBS approval letter DR ANGELA DRAMOWSKI N18.04.052	13/07/2018	1
Local Approval	14. NeoOBS PIL 12.07.18_V2.0	18/07/2018	2.0
Local Approval	17. NeoOBS ICF English+ China Final 19.07.18_V3.1-SCH-logo	19/07/2018	3.1
Local Approval	14. NeoOBS ICF Final 19.07.18_V3.1_7.9.18	19/07/2018	3.1
Local Approval	15. NeoOBS ICF Final 19.07.18_V3.1_wih DoW-latest mod_7.9.18-	19/07/2018	3.1
Local Approval	16. NeoOBS PIL FINAL 19.07.18_V2.1_7.9.18	19/07/2018	2.1
Local Approval	9. NeoOBS -South Africa-ICF Final 20.07.18_V3.1_Tygerberg	20/07/2018	3.1
Local Approval	10. NeoOBS -South Africa- PIL Final 20.07.18_V2.1_Tygerberg	20/07/2018	2.1
Local Approval	NeoOBS -South Africa-Stellenbosch amendment 23 JULY 2018	23/07/2018	1
Local Approval	10. NeoOBS_Italy_Ethics Approval V1.0 Estratto De Luca NeoOBS 001	30/07/2018	1.0
Local Approval	NeoOBS-Thailand-Official approval letter NeoOBS0001	09/08/2018	1
Local Approval	NeoOBS - Vietnam - _EC Approval_Vietnam_EN	10/08/2018	1
Local Approval	11. NeoOBS Ethical Approval letter - Lady Hardinge - Delhi - 05-09-2018	05/09/2018	1
Local Approval	11. NeoOBS - Amendment Approval - Italy	12/09/2018	1
Local Approval	NeoOBS_Bangladesh_ Ethics aproval_Amendment	16/09/2018	1
Local Approval	13. NeoObs Kilifi ICF V2.3 of 17 Sep 2018_English final	17/09/2018	2.3
Local Approval	NeoOBS_ China - Ethics approval -Beijing Children's Hospital	20/09/2018	1
Local Approval	NeoOBS_China - Ethics approval -Beijing-Children_s-Hospital - English Version	20/09/2018	1
Local Approval	9. NeoObs_Ethics approval_Greece_written Greek - Initial approval	20/09/2018	1
Local Approval	SGUL - NeoOBS REC Approval Letter for version 1 protocol	25/09/2018	1
Local Approval	11. NeoOBS -Uganda- ENG ICF signature page v 2.0 _ 17 Oct 2018	17/10/2018	1
Local Approval	13. NeoOBS -Uganda- Eng verbal consent for parents with very sick babies v 1.1 _ 17 Oct 2018	17/10/2018	1.1
Local Approval	14. NeoOBS -Uganda- PIS Eng Information sheet v 2.0 _ 17 Oct 18	17/10/2018	2.0
Local Approval	12. NeoOBS - Ethics approval - Translated into English Shenzhen	18/10/2018	1
Local Approval	13. NeoOBS - Ethics approval (part 1) Shenzhen	18/10/2018	1
Local Approval	14. NeoOBS - Ethics approval (part 2) Shenzhen	18/10/2018	1
Local Approval	15. NeoOBS - Ethics approval (part 3) Shenzhen	18/10/2018	1
Local Approval	11. NeoOBS - SERU No 3758_Approval - KEMRI, Kilifi County Hosptial	07/11/2018	1
Local Approval	10. NeoOBS - Ethical approval for amendment - Greece Amendment	23/11/2018	1
Local Approval	12.NeoOBS - India - Ethics Approval NeoOBS KEM Mumbai	12/12/2018	1
Local Approval	13. NeoOBS - India -PIL + ICD English NeoOBS V2.3	12/12/2018	2.3
Local Approval	12. NeoOBS - Ethics Approval - Beijing Obstetrics and Gynecology Hospital (part 1)	17/12/2018	1

Document Type	File Name	Date	Version
Local Approval	13. NeoOBS - Ethics Approval - Beijing Obstetrics and Gynecology Hospital (part 2)	17/12/2018	1
Local Approval	14. NeoOBS - Certificate clearance for no HGRAC Approval BOGH PART 1	17/12/2018	1
Local Approval	15. NeoOBS - Certificate clearance for no HGRAC Approval BOGH PART 2	17/12/2018	1
Local Approval	12. NeoOBS - OxTREC ethics approval - Kenya	03/01/2019	1
Local Approval	17. NeoOBS -Uganda- initial SOMREC Approval _ 15 Jan 2019 - Uganda	15/01/2019	1
Local Approval	16. NeoOBS - Certificate clearance for no HGRAC Approval SCH	11/02/2019	1
Local Approval	18. NeoOBS -Uganda- Mulago Admin Clearance Letter__Received 06 Mar 2019	06/03/2019	1
Local Approval	20. NeoOBS ICF English +China Final 19.07.18_V3.1_SCH-logo	18/07/2019	3.1
Local Approval	22. NeoOBS PIL English+China FINAL 19.07.18_V2.1-SCH-logo	18/07/2019	2.1
Local Approval	NeoOBS_Brazil_TCLE_PT_V3_julho19 (1)	19/07/2019	V3
Local Approval	16. NeoOBS_Brazil TCLE_Informed Consent Form FOR STORAGE OF BIOLOGICAL SAMPLES_v 1_1_20190729	29/07/2019	1.1
Local Approval	NeoOBS_Brazil_biorrepositorio_versao_1_1_20190729_Santa Casa	29/07/2019	1_1
Local Approval	NeoOBS_Brazil_TCLE_bebes_versao_1_3_20190729_Santa Casa	29/07/2019	1_3
Local Approval	15. NeoOBS_TCLE_Free and clarified consent form for parents legal guardians_Version_1_3_20190729	30/07/2019	1.3
Local Approval	12. NeoOBS - Brazil - CONEP approval - consent amendment 1.3 and bio samples 1.1	31/07/2019	1.1
Local Approval	NIMBi_Consent_110119	01/11/2019	0.3
Local Approval	NIMBi_UPenn_Approval_04212020	22/04/2020	1
Local Approval	Gambia_Consent_2020_v4.0	18/05/2020	4.0
Local Approval	SHARE_UPenn_Approval	09/06/2020	1
Local Approval	NIMBi_CHOP_Approval	25/07/2020	1
Local Approval	SHARE_HRDC_Approval	21/04/2022	1
Local Approval	SHARE_UB_Approval	11/05/2022	1
Local Approval	SHARE_PMH_Approval	20/05/2022	1
Local Approval	NIMBi_HRDC_Approval	15/02/2024	1
Local Approval	NIMBi_PMH_Approval_20032024	20/03/2024	1
Local Approval	BARNARDS_exampleConsentForm	27/05/2024	1
Local Approval	BARNARDS1-Ethical Approval	27/05/2024	1
Local Approval	Table v2 - Local Approvals	27/05/2024	2
Local Approval	NeoOBS_Brazil_PB_PARECER_CONSUBSTANCIADO_CEP_3514806_E1	19/08/2024	1

Any subsequent changes to the application must be submitted to the Committee via an Amendment form on the ethics online applications website: http://leo.lshtm.ac.uk .

Best of luck with your project.

Yours sincerely,

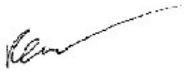

Rebecca Carter

Ethics Facilitator

Ethics@lshtm.ac.uk
http://www.lshtm.ac.uk/ethics/
